# A key loop in the catalytic pocket of the PL17 family of alginate lyases determines minimal substrate recognition

**DOI:** 10.1016/j.jbc.2025.110467

**Published:** 2025-07-09

**Authors:** Xue Li, Lanzeng Zhang, Yongqi Tang, Yi Li, Xinyu Wu, Yingjie Li, Lushan Wang

**Affiliations:** State Key Laboratory of Microbial Technology, Shandong University, Qingdao, China

**Keywords:** alginate lyase, oligosaccharide, oligo-alginate lyase pair, bacterial genetics, enzyme mechanism, minimal degrading substrate, loop swapping, structure-function

## Abstract

Alginate, a major component of brown macroalgae, is an alternative feedstock for biorefining. The degradation of alginate oligosaccharides (AOSs) is a key prerequisite for biorefining, which usually requires at least two oligo-alginate lyases (Oals). However, the function and minimal substrate recognition mechanisms of different Oals in alginate metabolism remain poorly understood. In this study, a pair of PL17 family Oals (*Va*Aly17A and *Va*Aly17B) was identified, which is universal in alginate-degrading *Vibrio* species. *Va*Aly17A is crucial for alginate metabolism, primarily acting on substrates larger than disaccharides, while *Va*Aly17B contributes to rapid alginate utilization by converting disaccharides into monomers. The distinct minimal degrading substrates of the two alginate lyases are determined by a critical loop, Loop1, around the active groove. *Va*Aly17A, with a shorter Loop1, forms an open groove for binding larger substrates, while the longer Loop1 in *Va*Aly17B results in a shorter catalytic cleft that accommodates only smaller substrates like disaccharides. Loop swapping experiments indicate that the shorter Loop1 is crucial for interacting with larger substrates, and structure alignment suggests that this loop may serve as a hallmark to distinguish the minimal substrates among PL17 Oals. Altogether, this study, for the first time, identifies a loop of PL17 Oals determining minimal substrate recognition and provides a new strategy for distinguishing the minimal recognition patterns of PL17 Oals.

Alginates are major components of the cell wall of brown seaweed, which can account for about 40% of the dry weight (1). They are composed of α-l-guluronic acid (G) and β-d-mannuronic acid (M) that could form three arranging types, including homopolymeric (polyG and polyM) and heteropolymeric (polyMG) blocks (2). In brown macroalgae, mannuronan C-5 epimerases (ManC5-Es) control the distribution pattern of G and M in alginates, leading to polysaccharide compositions and arrangements varying with species, tissue, season, or life cycle stage (3, 4, 5, 6). Different from algal alginates, the polysaccharides from microorganisms are usually acetylated on the O-2 and/or O-3 of β-d-mannuronate by mannuronate acetylase (7, 8).

As a renewable resource for sustainability, the degradation of alginates is cleaved by alginate lyases *via* the β-elimination reaction. To date, alginate lyases are categorized into 16 families of polysaccharide lyases (PLs) in the CAZy database, including PL5, PL6, PL7, PL8, PL14, PL15, PL17, PL18, PL31, PL32, PL34, PL36, PL38, PL39, PL41, and PL44 (9, 10). Among them, the PL32 enzyme is reclassified as PL_nc (not classified) due to insufficient diversity to create a family. Based on their cleavage specificity, alginate lyases are grouped as M-specific lyases (polyM lyase, EC 4.2.2.3), G-specific lyases (polyG lyase, EC 4.2.2.11), or bifunctional lyases (EC 4.2.2.-) (11, 12). Based on the catalysis mode of catalysis action, alginate lyases are classified into endo-type (EC 4.2.2.-) and exo-type lyases (EC 4.2.2.26). Most exo-type lyases are found to be oligo-alginate lyases (Oals), which are capable of degrading large oligosaccharides into small oligosaccharides and unsaturated monosaccharides. In addition, the crystal structures of alginate lyases from different families are grouped into three types: (α/α)_n_ toroid (PL5, PL15, PL17, PL38, and PL39), β-jelly roll (PL7, PL14, and PL18), and β-helix fold (PL6 and PL31) (13, 14).

During the alginate-utilizing process in most bacteria, alginates are first degraded into alginate oligosaccharides (AOSs) by alginate lyases. After being transported into the cell, AOSs are digested by Oals into unsaturated monosaccharides, 4-deoxy-l-*erythro*-4-hexenopyranuronate (Δ). Δ can be spontaneously or enzymatically converted into 4-deoxy-l-*erythro*-5-hexoseulose uronate (DEH) (15), which may undergo hydration and further be cyclized into two epimers of a 5-member hemiketal, called 4-deoxy-d-*manno*-hexulofuranosidonate (DHF) (16, 17). DEH is reduced by DEH reductase to form 2-keto-3-deoxy-gluconate (KDG), and KDG is stepwise converted into glyceraldehyde triphosphate (G3P) and pyruvate *via* the Entner-Doudoroff (ED) pathway (18). By engineering the metabolic pathway of pyruvate and its product acetyl-CoA, alginate together with mannitol can be degraded into several biochemical products, such as ethanol (19, 20, 21), 2,3-butanediol (22), lycopene (22), and citramalate (23). Except for the marine bacterium *Falsirhodobacter* sp. alg1 that only uses one endo lyase and one Oal to achieve efficient degradation of alginate (24), most microorganisms require complex alginolytic systems, which harbor up to 15 alginate lyases and up to 5 Oals, for complete depolymerization of alginate (18, 25). It has been suggested that multiple alginate lyases usually have different component compositions, action patterns, and product distributions, which together allow the bacterium to fully digest alginate polymers into AOSs (26, 27, 28, 29). However, little is known about the necessity of complex Oal systems for AOS degradation.

*Vibrio alginolyticus* ATCC 17749 was isolated from spoiled horse mackerel and is capable of fast growth by using alginate as the sole carbon source. A growth rate similar to that of the conventional host *Escherichia coli* was observed in LB medium (Fig. S1*A*). In addition, *V. alginolyticus* ATCC 17749 shows strong tolerance to high osmotic pressure and can quickly grow in the presence of up to 12.5% NaCl (Fig. S1*B*). Therefore, *V. alginolyticus* ATCC 17749 has great potential to be an efficient microbial platform for brown algae bioconversion. Understanding the process of AOS metabolism is a prerequisite to the efficient biorefining of alginate by genetic engineering methods. In this study, we set out to distinguish the role of a PL17 Oal pair (denoted as *Va*Aly17A and *Va*Aly17B) in *V*. *alginolyticus* ATCC 17749 by comparing their functions for alginate utilization *in vivo* and *in vitro*. Our data indicated that, in *V. alginolyticus* ATCC 17749, the PL17 family Oal pair is essential for the stepwise degradation of AOSs. A loop named Loop1 around the active groove is demonstrated to be the key factor leading to the different minimal degrading substrates of *Va*Aly17A and *Va*Aly17B. Structural comparisons suggest this loop could serve as a hallmark to distinguish the minimal degrading substrates of different PL17 Oals.

## Results

### A PL17 Oal pair occurs in alginate-degrading *Vibrio* species

**Figure 1:**
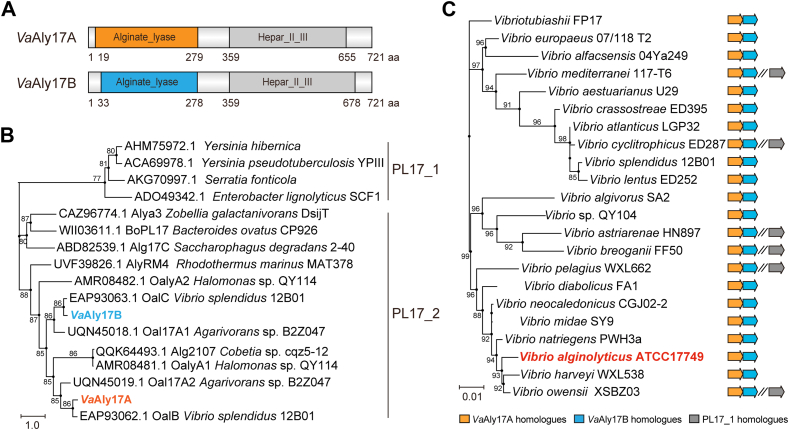
**Sequence analysis of the PL17 Oal pair.***A*, modular organization of *Va*Aly17A and *Va*Aly17B from *V. alginolyticus* ATCC 17749. *B*, phylogenetic analysis of *Va*Aly17A and *Va*Aly17B. *C*, identification of the PL17 Oal pair in alginate-degrading *Vibrio* species. All alginate-degrading *Vibrio* species in the CAZy database (Last update: 2024-05-24) were analyzed, and only one representative strain was selected for the construction of the phylogenetic tree when more than two strains belonging to the same species were present. The 16S rRNA gene was used to obtain the tree. The phylogenetic trees were constructed using the MEGA 11 software *via* the neighbor-joining method. A bootstrap analysis of 1000 replicates was conducted.

**Figure 2:**
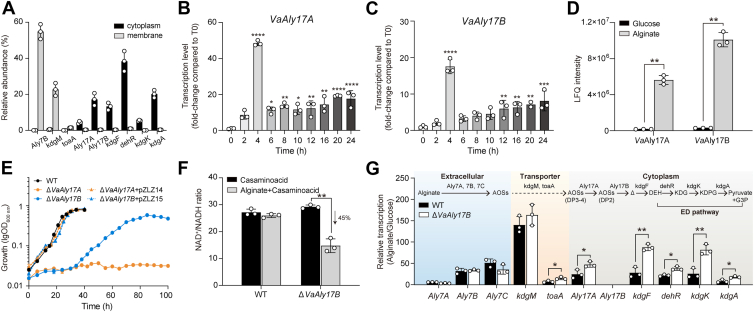
**Characterization of the physiological functions of *Va*Aly17A and *Va*Aly17B *in vivo*.***A*, relative abundance of alginate degradation related proteins in cell membrane and cytoplasm samples of *V*. *alginolyticus* ATCC 17749 with 0.2% sodium alginate. Transcription of *VaAly17A* (*B*) and *VaAly17B* (*C*) in the WT cultured in alginate medium during the whole growth. Values are indicated as fold changes relative to the pre-culture in glucose medium, which is also used as the inoculum (T0). *D*, proteomic analysis of *Va*Aly17A and *Va*Aly17B. Cells for proteomic analysis were grown to the stationary phase in the presence of alginate and glucose, respectively. LFQ, label-free quantitation. *E*, growth of WT, mutant strains Δ*VaAly17A* and Δ*VaAly17B*, and complemented strains Δ*VaAly17A* + pZLZ14 and Δ*VaAly17B* + pZLZ15 with their respective plasmids. For the growth assay in alginate medium, cells were cultured in glucose medium and used as the inoculum to grow with alginate as the sole carbon source. *F*, cellular NAD^+^/NADH ratio assays of WT and Δ*VaAly17B* mutant. The contents of NAD^+^ and NADH were detected in the exponential phase cells grown in M9 high salt medium supplemented with 0.2% casamino acid in the presence of alginate or its absence and normalized by the luminescence signal. *G*, effect of the *VaAly17B* deletion on the expression of genes involved in alginate metabolism. Cells at an early exponential phase in glucose or alginate medium were used for transcription analysis. Based on bioinformatic analysis data, a full pathway is suggested in *V. alginolyticus* ATCC 17749 as follows: Alginates are first converted into AOSs by membrane-anchored PL7 alginate lyases, including *Va*Aly7A (Aly7A), *Va*Aly7B (Aly7B), and *Va*Aly7C (Aly7C). Once transferred into the cytoplasm *via* a conserved porin KdgM and inner membrane symporter ToaA (18, 19), AOSs larger than disaccharides are catalyzed into disaccharides by *Va*Aly17A (Aly17A). Subsequently, *Va*Aly17B (Aly17B) degrades disaccharides produced by *Va*Aly17A or extracellular alginate lyases into unsaturated monomers. The unsaturated monomer (Δ) can spontaneously isomerize or be converted into linear ketonized forms DEH *via* KdgF catalysis, which enters the ED pathway and is subsequently converted into pyruvate and G3P. Values represent the mean ± standard deviation of three independent biological replicates. Statistical comparisons were performed using an unpaired two-tailed Student’s *t* test. Significance levels are indicated as follows: *p* < 0.05 (∗), *p* < 0.01 (∗∗), *p* < 0.001 (∗∗∗), *p* < 0.0001 (∗∗∗∗).

Bioinformatic analysis revealed that *V. alginolyticus* ATCC 17749 harbors two tandem-located genes coding for two PL17 family Oals, named *Va*Aly17A (GenBank accession number: AGV20268.1) and *Va*Aly17B (GenBank accession number: AGV20269.1), respectively. *Va*Aly17A and *Va*Aly17B have similar domain compositions, one annotated as the PL17 alginate lyase domain and the other as the Hepar_Ⅱ_Ⅲ_C superfamily domain (Pfam: PF07940) (Fig. 1*A*). *Va*Aly17A shows the highest similarity of 84% with oligo-alginate lyase OalB from *Vibrio splendidus* 12B01, and *Va*Aly17B has the highest similarity of 88% with oligo-alginate lyase OalC from *V. splendidus* 12B01 (Fig. 1*B*). *Va*Aly17A and *Va*Aly17B share a similarity of 63%. Since no putative signal peptide was predicted using the SignalP 6.0 server, *Va*Aly17A and *Va*Aly17B are likely located in the cytoplasm. Consistent with this, LC-MS/MS analysis revealed that *Va*Aly17A and *Va*Aly17B were mainly observed in the cytoplasm (Fig. 2*A*). Unexpectedly, all alginate-degrading *Vibrio* species in the CAZy database contain a similar gene organization, and the tandem-located PL17 Oal pair seems to be located on the same operon (Fig. 1*C*). The pair of alginate lyases belongs to the PL17_2 subfamily (Fig. 1*B*). Among these microorganisms, some *Vibrio* species also contain one more PL17 Oal, whereas it is not clustered with the PL17 pair. This clade of alginate lyases belongs to the PL17_1 subfamily (Fig. 1*C*). Further sequence alignment revealed that, compared to *Va*Aly17A and its homologues, *Va*Aly17B and its homologs contain an addition sequence insertion between sheets β3 and β4 (Fig. S2). Taken together, our bioinformatic analysis suggested that alginate-degrading *Vibrio* species usually contain at least a pair of PL17 Oals, which is tandem-located in the genome.

### *Va*Aly17A, but not *Va*Aly17B, is required for the growth of alginate

To understand their role in AOS degradation, the expression of *VaAly17A* and *VaAly17B* was first investigated during the whole growth process in the presence of alginate. As shown in Figure 2, *B* and *C*, the transcription of *VaAly17A* and *VaAly17B* was quickly induced by alginate when WT cells were inoculated from glucose medium to alginate medium. Both *VaAly17A* and *VaAly17B* exhibited the greatest expression levels after 4 h of cultivation, followed by a sharp reduction. Then, the expression of *VaAly17A* and *VaAly17B* slowly increased over time. Consistent with the transcription data, the proteomic comparison of the WT strain grown in the presence of alginate and its absence demonstrated that the expression of *Va*Aly17A and *Va*Aly17B is significantly upregulated by alginate (Fig. 2*D*). Therefore, *Va*Aly17A and *Va*Aly17B display an alginate-dependent expression.

To distinguish the physiological functions of *Va*Aly17A and *Va*Aly17B on AOS metabolism, two mutant strains were constructed by DNA homologue recombination and named Δ*VaAly17A* and Δ*VaAly17B*, respectively. When grown in M9 medium with 0.2% sodium alginate as the sole carbon source, WT cells reached the stationary phase after 36 h of incubation (Fig. 2*E*). However, the loss of *VaAly17A* led to no growth in alginate medium, suggesting that *Va*Aly17A is required for alginate utilization. When the Δ*VaAly17A* mutant was complemented with a WT *VaAly17A* allele, cells restored to WT-like growth in alginate medium (Fig. 2*E*). Differently, the Δ*VaAly17B* strain displayed an extended lag phase and reached its stationary phase about 50 h later than the WT cells. The final cell density of the Δ*VaAly17B* strain was reduced in alginate medium (Fig. 2*E*). Complementation of *VaAly17B* with the WT allele restored its growth with alginate back to the WT levels. Altogether, our data suggested that *Va*Aly17A and *Va*Aly17B play distinct roles in alginate metabolism: *Va*Aly17A is essential for alginate utilization, while *Va*Aly17B is required for the fast growth on alginate.

### *Va*Aly17A and *Va*Aly17B exhibit different biochemical characteristics *in vitro*

#### Protein production and purification

To further uncover their different roles in AOS metabolism, *Va*Aly17A and *Va*Aly17B were heterologously produced in *E. coli* BL21 (DE3) and purified. The molecular weights of *Va*Aly17A and *Va*Aly17B on the SDS-PAGE were about 80 kDa, which are in line with their respective theoretical molecular weights of 81,525 Da and 81,023 Da (Fig. S3). The purified protein yields of *Va*Aly17A and *Va*Aly17B were about 3.5 mg per 1 L of LB culture and 28.8 mg per 1 L of LB culture, respectively.

#### Effect of temperature and pH

**Figure 3:**
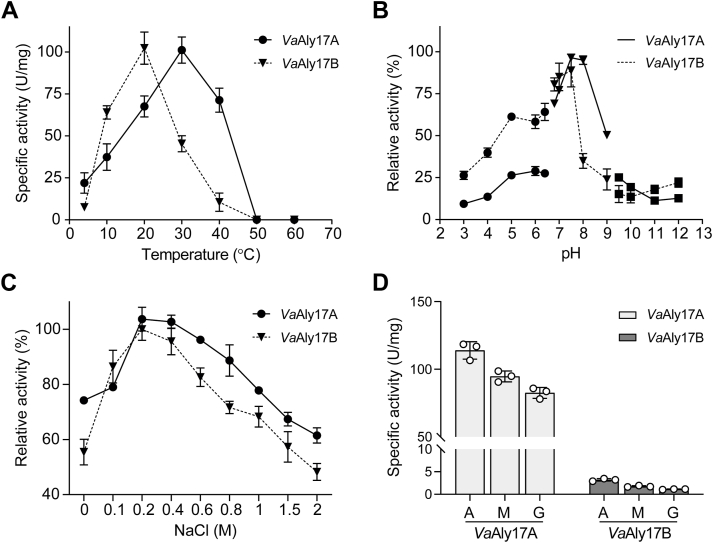
**Biochemical characteristics analysis of *Va*Aly17A and *Va*Aly17B.***A*, optimal temperature analysis. The reaction was performed in 50 mM Tris-HCl (pH 8.0, 200 mM NaCl) at different temperatures. *B*, optimal pH analysis. The reaction was performed at different pHs, and the reaction temperatures were 30 °C and 20 °C for *Va*Aly17A and *Va*Aly17B, respectively. Buffer pH 3.0 to 6.4 was prepared from 50 mM citrate-citric acid; Buffer pH 6.8 to 9.0 is prepared from 50 mM Tris-HCl; Buffer pH 9.5 to 12.0 is prepared from 50 mM glycine-NaOH. *C*, Effect of NaCl on the activity of *Va*Aly17A and *Va*Aly17B. The reaction was performed in 50 mM Tris-HCl (pH 8.0) and NaCl concentrations ranging from 0 to 2.0 M at the optimum temperature. *D*, Substrate specificities of *Va*Aly17A and *Va*Aly17B toward sodium alginate (A), polyM (M), and polyG (G), respectively. *Va*Aly17B showed little activity toward these polymers, and its activities toward alginate, polyM, and polyG were 3.2 ± 0.3 U/mg, 1.8 ± 0.1 U/mg, and 1.1 ± 0.1 U/mg, respectively. Results were obtained from three independent experiments. Values are shown as means ± standard deviations.

*Va*Aly17A had the greatest activity at 30 °C, while *Va*Aly17B showed the optimal activity at 20 °C (Fig. 3*A*). When the temperature was greater than 40 °C, little activity of *Va*Aly17B was observed, while more than 70% activity of *Va*Aly17A was observed. When examined at different pH values, *Va*Aly17A and *Va*Aly17B displayed the maximum activity at pH 7.5 to 8.0 (Fig. 3*B*), which is similar to PL17 OalB and OalC from *V. splendidus* 12B01 (30), Alg17B from marine strain BP-2 (31), and OalV17 from *Vibrio* sp. SY01 (32). These data indicated that both *Va*Aly17A and *Va*Aly17B are medium-temperature and alkaline alginate lyases, and *Va*Aly17B is more sensitive to high temperatures compared to *Va*Aly17A.

#### Effect of NaCl

Since *V. alginolyticus* ATCC 17749 can grow fast in the presence of up to 12.5% NaCl, its membrane-anchored PL7 alginate lyase *Va*Aly7B did not have any activity when NaCl was absent (33). Therefore, to investigate the effect of NaCl on enzyme function, the activity of *Va*Aly17A and *Va*Aly17B was examined at different NaCl concentrations. As shown in Figure 3*C*, *Va*Aly17A and *Va*Aly17B retained more than 70% and 50% of activity when NaCl was omitted. *Va*Aly17A and *Va*Aly17B showed the highest activity when NaCl concentration was 200 mM, and when 2 M NaCl was present, > 60% activity of *Va*Aly17A and > 40% activity of *Va*Aly17B were retained, respectively. Therefore, *Va*Aly17A and *Va*Aly17B are salt-tolerant.

#### Specific activities toward different substrates

*Va*Aly17A displayed the greatest specific activity toward alginate (114 U/mg), followed by polyM (95 U/mg) and polyG (83 U/mg). A similar pattern was also observed in *Va*Aly17B, while its activities toward these polymers were much lower than those in *Va*Aly17A, with a specific activity of 1.1 to 3.2 U/mg (Fig. 3*D*). The significantly different alginolytic activities of *Va*Aly17A and *Va*Aly17B toward polymers may be closely related to their physiological functions *in vivo*.

#### Enzyme kinetics parameters of VaAly17A and VaAly17B

The kinetic parameters of *Va*Aly17A and *Va*Aly17B were measured with increasing concentrations of alginate, polyM, and polyG ranging from 0.01 to 3 mg/ml. As shown in Table 1, *Va*Aly17A exhibited *K*_m_ values of 0.010, 0.011, and 0.008 mg/ml toward alginate, polyM, and polyG, respectively, indicating a greater binding of polyG. *Va*Aly17B showed *K*_m_ values of 0.022, 0.021, and 0.020 mg/ml. The *K*_m_ values of *Va*Aly17A toward three substrates were approximately 50% of those of *Va*Aly17B, suggesting that *Va*Aly17A has greater substrate-binding affinities toward these polymer substrates. The *k*_cat_ values of *Va*Aly17A toward three polymers were similar, indicating similar rates of product formation. A similar pattern was also observed in *Va*Aly17B, whose *k*_cat_ values were similar when alginate, polyM, and polyG were used as the substrates (Table 1). However, the *k*_cat_ values of *Va*Aly17A for alginate, polyM, and polyG were approximately eight times higher than those of *Va*Aly17B, suggesting that *Va*Aly17A has a faster rate of formation of product. The *k*_cat_/*K*_m_ ratios of *Va*Aly17A toward these polysaccharides were significantly greater than those of *Va*Aly17B, indicating that *Va*Aly17A has greater catalytic efficiencies toward three substrates.

**Table 1:** Kinetic parameters of *Va*Aly17A and *Va*Aly17B toward different alginate substrates

Parameter	Sodium alginate	PolyM	PolyG
*Va*Aly17A			
*V*_max_ (U mg of protein^−1^)	59.2 ± 1.7	55.9 ± 2.0	47.3 ± 1.9
*K*_m_ (mg mL^−1^)	0.010 ± 0.001	0.011 ± 0.002	0.008 ± 0.001
*k*_cat_ (s^−1^)	0.080 ± 0.002	0.076 ± 0.003	0.064 ± 0.003
*k*_cat_/*K*_m_ (mg^−1^ ml s^−1^)	8.04	6.91	8.03
*Va*Aly17B			
*V*_max_ (U mg of protein^−1^)	7.2 ± 0.3	6.3 ± 0.3	5.7 ± 0.2
*K*_m_ (mg mL^−1^)	0.022 ± 0.003	0.021 ± 0.003	0.020 ± 0.003
*k*_cat_ (s^−1^)	0.010 ± 0.0004	0.009 ± 0.0004	0.008 ± 0.0003
*k*_cat_/*K*_m_ (mg^−1^ ml s^−1^)	0.44	0.41	0.39

Taken together, biochemical data *in vitro* indicated that *Va*Aly17A and *Va*Aly17B are medium-temperature, alkaline, and salt-tolerant alginate lyases, and the major difference is their activity toward polymer alginate substrates, which may result in distinct physiological functions on alginate metabolism *in vivo*.

### A stepwise degradation of AOSs is achieved by *Va*Aly17A and *Va*Aly17B

**Figure 4:**
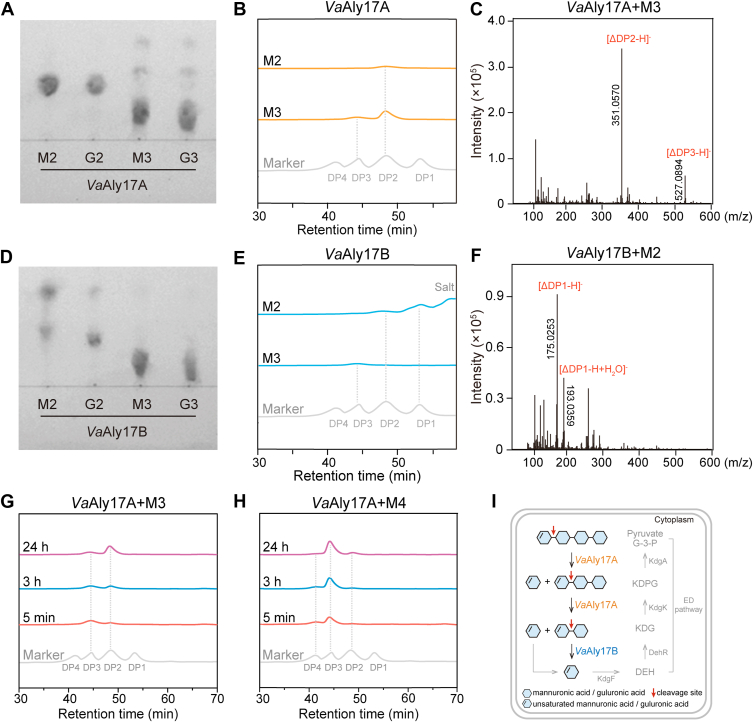
**Minimal substrate and catalysis mode analysis of *Va*Aly17A and *Va*Aly17B.***A* and *D*, TLC analysis for the minimal substrates of *Va*Aly17A and *Va*Aly17B. M-type and G-type AOSs were used, and similar catalytic patterns were observed with M- and G-type AOSs. *B*, gel filtration HPLC analysis of AOSs produced by *Va*Aly17A. Since UV at 235 nm was used to detect unsaturated product, the substrate used was mainly saturated AOS. Therefore, when no degradation occurs, no peak is observed by HPLC (64). For example, when trisaccharides were used as the substrate, *Va*Aly17A could catalyze MMM into M (saturated monomers) and ΔM (unsaturated disaccharides). Therefore, only unsaturated disaccharides were detected by HPLC. *C*, ESI-MS analysis of the AOSs produced by *Va*Aly17A. When M3 was used as the substrate, M2 and M3 were identified after 24 h of reaction. When M2 was used as the substrate, only AOSs with DP2 were identified (data not shown). *E*, gel filtration HPLC analysis of AOSs produced by *Va*Aly17B. *Va*Aly17B could not degrade M3, and no peak was observed when M3 was used as the substrate. When M2 was used as the substrate, the unsaturated monomer was observed. In addition, a peak proposed for salt was also present (65). *F*, ESI-MS analysis of the product produced by *Va*Aly17B. When M2 was used as the substrate, two types of monomers were identified. When M3 was used as the substrate, only AOSs with DP3 were identified (data not shown). *G*, product analysis of *Va*Aly17A reacting with M3 over time. Since a double bond is produced in the sugar by alginate lyases, which is on the reducing end of the cleaved glycosidic bond, the product obtained from this side will contain an unsaturated sugar at the reducing end of the AOS chain. Using the substrate of the saturated M-unit trimer as an example, this reaction could have two outcomes as follows: MM + Δ (degradation from the reducing end of M3) or M + ΔM (degradation from the nonreducing end of M3). Based on this, *Va*Aly17A is proposed to degrade the substrate from the nonreducing end of the sugar chain because the yields of unsaturated disaccharides (ΔM) are gradually increased over time. *H*, product analysis of *Va*Aly17A reacting with M4 over time. Results were obtained from representative experiments. *I*, The degradation process of AOSs in the cytoplasm of *V. alginolyticus* ATCC 17749.

To figure out the reason why *Va*Aly17A and *Va*Aly17B play different roles in AOS metabolism in *V. alginolyticus* ATCC 17749, the minimal degrading substrates of *Va*Aly17A and *Va*Aly17B were examined by using M-type and G-type of oligomers with different degrees of polymerization (DPs). As shown in Figure 4*A*, *Va*Aly17A could not catalyze disaccharides into monomers while degrading trisaccharides into smaller products, suggesting that the minimal degrading substrate of *Va*Aly17A is a trisaccharide. Differently, *Va*Aly17B could obviously degrade disaccharides into unsaturated monomer and equimolar amount of saturated monomer, whereas degradation was hardly observed when trisaccharides were used as the substrate (Fig. 4*D*), indicating that *Va*Aly17B in *V. alginolyticus* ATCC 17749 mainly catalyzes disaccharides into monomers. In addition, high performance liquid chromatography (HPLC) and electrospray ionization mass spectrometry (ESI-MS) were performed to confirm the saccharides produced by *Va*Aly17A and *Va*Aly17B. Since thin layer chromatography (TLC) data showed that *Va*Aly17A and *Va*Aly17B have similar minimal substrate recognition patterns with M- and G-type AOSs, only M-type substrates were used for HPLC and ESI-MS analysis. In agreement with TLC data, when *Va*Aly17A reacted with M2, little product was detected, whereas unsaturated M2 was produced when M3 was used as the substrate (Fig. 4, *B* and *C*). *Va*Aly17B could degrade M2 into monomers but could not obviously catalyze the substrate of M3 (Fig. 4, *E* and *F*). In addition, *Va*Aly17B could also act on unsaturated disaccharides (Fig. S4). HPLC data also revealed that both *Va*Aly17A and *Va*Aly17B are exolytic, a conserved action mode observed in Oal families (10). Furthermore, the action mode of *Va*Aly17A was also investigated with M3 and M4 as substrates. As shown in Figure 4, *G* and *H*, *Va*Aly17A could degrade the substrate larger than disaccharide from the nonreducing end because the yields of unsaturated disaccharide ΔM gradually increased over time.

To further distinguish the roles of *Va*Aly17A and *Va*Aly17B in alginate metabolism, the specific activities of *Va*Aly17A and *Va*Aly17B toward alginate oligosaccharides with different degrees of polymerization (M2, M3, M4, M5, M6, G2, G3, G4, G5, and G6) were examined. As shown in Table 2, the activities of *Va*Aly17A increased as the degrees of polymerization increased. *Va*Aly17A exhibited the greatest activity toward M-type hexasaccharides. The activities of *Va*Aly17A toward M-type oligosaccharides were much greater than those toward G-type oligosaccharides. Different from *Va*Aly17A preferring large AOSs, *Va*Aly17B showed the greatest activity toward M-type disaccharides, while the activity toward G-type disaccharides was about 20% of that toward M-type disaccharides. When the substrate was larger than disaccharides, the activity of *Va*Aly17B was significantly decreased (Table 2). Based on these data, a stepwise reaction is proposed as follows: once AOSs are transferred into the cytoplasm, *Va*Aly17A first degrades AOSs larger than disaccharides into disaccharides and monomers, and subsequently, unsaturated dimers are catalyzed into unsaturated monomers Δ by *Va*Aly17B. Δ can spontaneously isomerize or be converted into linear ketonized forms DEH *via* KdgF catalysis, which enters the ED pathway and is subsequently converted into pyruvate and G3P (Fig. 4*I*).

**Table 2:** Substrate specificities of *Va*Aly17A and *Va*Aly17B toward different AOSs

Substrate	Mean specific activity (U/mg) ± SD	
	*Va*Aly17A	*Va*Aly17B
Dimer-M2	0.04 ± 0.10	12.14 ± 0.33
Dimer-G2	0.03 ± 0.01	2.77 ± 0.03
Trimer-M3	0.24 ± 0.03	0.06 ± 0.01
Trimer-G3	0.09 ± 0.05	0.05 ± 0.01
Tetramer-M4	0.61 ± 0.16	0.04 ± 0.01
Tetramer-G4	0.17 ± 0.10	0.05 ± 0.01
Pentamer-M5	22.24 ± 0.30	0.79 ± 0.03
Pentamer-G5	14.70 ± 0.44	0.26 ± 0.01
Hexamer-M6	39.38 ± 0.96	1.03 ± 0.05
Hexamer-G6	26.25 ± 1.08	0.73 ± 0.02

### Length of a loop around the active groove determines the minimum recognition patterns of *Va*Aly17A and *Va*Aly17B

**Figure 5:**
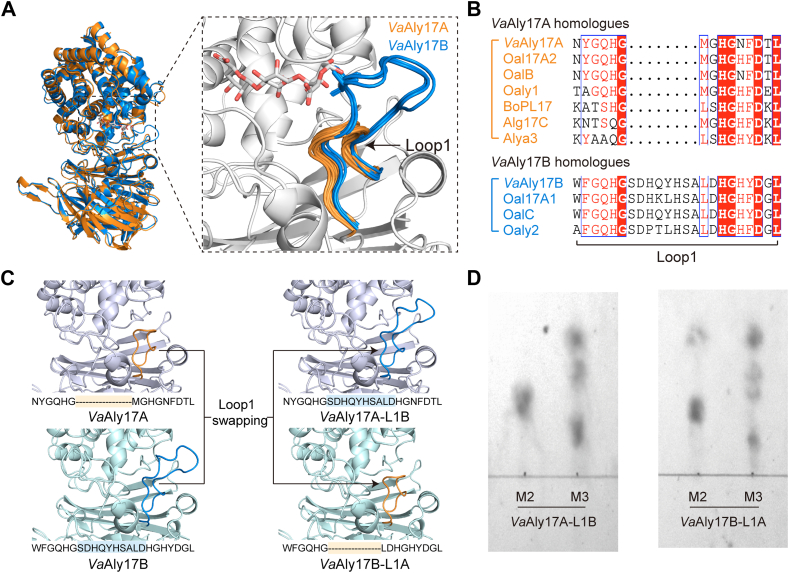
**Sequence and structure analysis of Loop1 and its swapping between *Va*Aly17A and *Va*Aly17B.***A*, structure alignment of *Va*Aly17A and *Va*Aly17B. The size of the loop named Loop1 in the active groove is obviously different between *Va*Aly17A and *Va*Aly17B. For structure alignment, *Va*Aly17A, *Va*Aly17B, and 28 other characterized PL17 Oals were used. Protein structures used for alignment were predicted with AlphaFold2. *B*, sequence alignment of Loop1 in characterized PL17 Oals. More details containing all characterized PL17 Oals are shown in Fig. S9. *C*, the schematic diagram of the loop swapping. The short Loop1 of *Va*Aly17A was changed to the respective long Loop1 of *Va*Aly17B, and *vice versa*. The *Va*Aly17A with the long Loop1 of *Va*Aly17B was named *Va*Aly17A-L1B, and the *Va*Aly17B with the short Loop1 of *Va*Aly17A was named *Va*Aly17B-L1A. The amino acid sequences of Loop1 for loop swapping are shown. The *yellow* and *blue* shading represent the exchanged portions of the short Loop1 and the long Loop1, respectively. *D*, TLC analysis for the minimal substrates of *Va*Aly17A-L1B and *Va*Aly17B-L1A. M-type AOSs (M2 and M3) were used as substrates, and the reaction was carried out for 24 h.

To uncover the mechanism leading to the different minimal degrading substrates of *Va*Aly17A and *Va*Aly17B, molecular docking analysis was carried out using AutoDock Vina. First, the overall structures of *Va*Aly17A and *Va*Aly17B were produced by AlphaFold2. At first glance, the predicted architectures of *Va*Aly17A and *Va*Aly17B are similar, consisting of an N-terminal α-barrel and a C-terminal β-sheet (Fig. 5*A*). The substrate is suggested to be bound between the α-barrel and the β-sheet (13, 34). The crystal structure of the AlyA3-G3 complex (PDB: 7BM6) (35) suggested that Asn^199^ and His^200^ can stabilize the negative charge and lower the p*K*a of the C5 proton at the +1 subsite, which are conserved among the PL17 family enzymes (Figs. S5 and S6). Structure alignment also showed that amino acid residues Tyr^429^ and Tyr^230^ in *Va*Aly17A and Tyr^441^ and Tyr^237^ in *Va*Aly17B are identical to the catalytic base and the catalytic acid in the crystal structures of BoPL17 (PDB: 8BDD) (36), AlyA3 (PDB: 7BJT) (35), Alg17C (PDB: 4NEI) (37), and AlgL17 (PDB: 9IRQ) (38). Therefore, they are proposed to function as the catalytic base and the catalytic acid, respectively. Consistent with the sequence alignment result that one additional sequence insertion is present in *Va*Aly17B (Figs. S2 and 5*B*), structure alignment data suggested that one loop named Loop1 in the active groove is obviously longer in *Va*Aly17B compared to *Va*Aly17A. This difference was also observed between *Va*Aly17A homologs and *Va*Aly17B homologues present in alginate-degrading *Vibrio* species (Fig. S7).

Molecular docking data of the *Va*Aly17A-MMG complex showed that the shorter Loop1 in *Va*Aly17A leads to a comparatively open groove, allowing the binding of longer substrates (Fig. S8*A*). However, the longer Loop1 in *Va*Aly17B occupies one end of the active site, resulting in the active groove being shorter than that in *Va*Aly17A. Therefore, only a small substrate, such as disaccharide, can bind to the groove in *Va*Aly17B (Fig. S8*B*). A close-up view of the active groove showed that the composition of candidate amino acid residues interacting with the substrate is similar at the −1 subsite in *Va*Aly17A and *Va*Aly17B (Fig. S8, *A* and *B*). However, compared to *Va*Aly17B, fewer amino acid residues in *Va*Aly17A were observed to interact with the sugar ring at the +1 subsite. This difference may result in the incapability of disaccharide degradation of *Va*Aly17A because of the weak binding of the substrate. Moreover, the residues at the +2 subsite are also distinct between *Va*Aly17A and *Va*Aly17B (Fig. S6), which is likely caused by their different sizes of Loop1. Therefore, *Va*Aly17A could degrade AOSs larger than disaccharides. Differently, *Va*Aly17B lacks the amino acid residues that interact with the sugar ring at the +2 subsite, which results in a preference for catalyzing disaccharides (Fig. S8*B*). Therefore, our bioinformatic data suggested that the length of Loop1 in the active groove determines the minimum recognition patterns of *Va*Aly17A and *Va*Aly17B.

**Figure 6:**
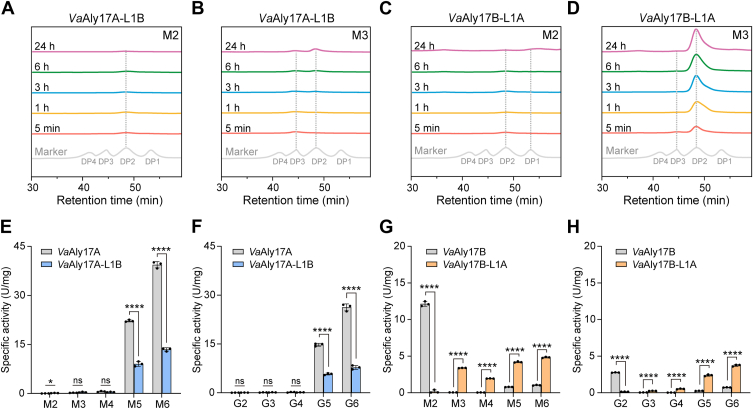
**Minimal substrate analysis and specific activity of *Va*Aly17A, *Va*Aly17B, *Va*Aly17A-L1B, and *Va*Aly17B-L1A.***A* and *B*, gel filtration HPLC analysis of sugars produced by *Va*Aly17A-L1B toward disaccharides (*A*, M2) and trisaccharides (*B*, M3) over time. *C* and *D*, gel filtration HPLC analysis of sugars produced by *Va*Aly17B-L1A toward disaccharides (*C*, M2) and trisaccharides (*D*, M3) over time. *E and F*, specific activity of *Va*Aly17A and *Va*Aly17A-L1B toward M-type AOSs (M2–M6, *E*) and G-type AOSs (G2–G6, *F*). *G* and *H*, specific activity of *Va*Aly17B and *Va*Aly17B-L1A toward M-type AOSs (M2–M6, *G*) and G-type AOSs (G2–G6, *H*). Data are presented as mean ± standard deviation from three independent biological replicates. Statistical significance was assessed using an unpaired two-tailed Student’s *t* test. *p* < 0.05 (∗), *p* < 0.01 (∗∗), *p* < 0.001 (∗∗∗), *p* < 0.0001 (∗∗∗∗).

To verify our bioinformatic hypothesis, loop swapping was further performed, and the schematic diagram was shown in Figure 5*C*. When the short Loop1 of *Va*Aly17A was replaced with the long Loop1 of *Va*Aly17B (*Va*Aly17A-L1B), few disaccharides could be degraded (Figs. 5*D* and 6*A*), but trisaccharides were catalyzed into dimers and monomers (Figs. 5*D* and 6*B*). *Va*Aly17B with the short Loop1 of *Va*Aly17A (*Va*Aly17B-L1A) exhibited reduced activity toward disaccharides (Fig. 6*C*) but restored the capability of trisaccharide degradation (Figs. 5*D* and 6*D*). In addition, the specific activities of the loop-swapped mutants *Va*Aly17A-L1B and *Va*Aly17B-L1A toward oligosaccharides with different degrees of polymerization were measured. As shown in Figure 6*E*, *F*, and Table S1, the activities of *Va*Aly17A-L1B toward pentasaccharides and hexasaccharides were significantly decreased, reaching only 41% (M5), 40% (G5), 34% (M6), and 30% (G6) of the wild-type activity levels. However, compared to the *Va*Aly17B, *Va*Aly17B-L1A with a short Loop1 exhibited poor activities toward dimers but significantly greater activities toward trimer, tetramer, pentamer, and hexamer oligosaccharides (Fig. 6*G*, *H*, and Table S1). These data suggested that the short Loop1 is critical for the *Va*Aly17A effective reaction with sugars larger than disaccharides. However, the loop-exchanged enzyme *Va*Aly17A-L1B was still unable to degrade disaccharides (Figs. 5*D* and 6*A*). This may be due to the weak interaction of *Va*Aly17A-L1B with the sugar, because fewer amino acid residues of *Va*Aly17A-L1B were observed for the binding of the sugar ring at the +1 subsite, compared to those in the wild-type *Va*Aly17B (Fig. S8, *B* and *C*). Therefore, our data demonstrated that the longer Loop1 in the active groove is the key factor preventing the reaction of *Va*Aly17B with sugars larger than disaccharides. Taken together, our loop-swapping results verified that, in *Va*Aly17A and *Va*Aly17B, the length of Loop1 in the active groove serves as the determinant for the recognition of the minimum degrading substrate.

Then, we wondered whether all PL17 family Oals feature varied sizes of Loop1 to determine the minimal substrate length for binding and catalyzing. To this end, the structures of all characterized PL17 Oals were predicted with AlphaFold2. As shown in Figure 5*A*, only two types of Loop1 were found, including a *Va*Aly17A-like Loop1 and a *Va*Aly17B-like Loop1. In line with this, Oals containing a *Va*Aly17A-like Loop1 prefer to catalyze AOSs larger than disaccharides (32, 34, 37, 39, 40, 41, 42). Three reported Oals, including OalC from *V. splendidus* 12B01 (30), Oaly2 from *Halomonas* sp. (43), and Oal17A1 from *Agarivorans* sp. B2Z047 (29), contain a *Va*Aly17B-like Loop1. Among them, OalC and Oal17A1 have been characterized as preferring to digest disaccharides (29, 30). Altogether, structure alignment data suggested that, in the PL17 Oals, the Loop1 in the active pocket may be a key factor in determining the size of the binding and degrading substrate. The two different types of Loop1 are not merely present in the PL17 Oal pair in alginate-degrading *Vibrio* species but are also conserved in all identified PL17 Oals. Therefore, the size of Loop1 can serve as a hallmark to predict the minimal substrate of different PL17 Oals.

### *Va*Aly17B is important to eliminate accumulated disaccharides and maintain intracellular redox homeostasis

As shown above, *Va*Aly17B is responsible for the digestion of alginate disaccharides, a type of acidic saccharide. Therefore, it is proposed that the accumulation of alginate disaccharides may affect intracellular redox homeostasis. For this reason, the intracellular redox conditions in WT and Δ*VaAly17B* mutant were compared by determining the ratio of NAD^+^ (oxidized)/NADH (reduced), which is an important redox couple for multiple redox reactions (44). As shown in Figure 2*F*, no obvious difference in the ratio of NAD^+^/NADH in the WT cells was found between the presence of alginate and its absence. However, when *VaAly17B* was deleted, the ratio of NAD^+^/NADH in the presence of alginate significantly decreased compared to that in the absence of alginate. This indicated that, compared to the WT strain, the accumulation of alginate acidic disaccharides led to a more reduced redox state in the Δ*VaAly17B* strain. Moreover, the accumulation of disaccharides could act as a signal to enhance the expression of genes involved in intracellular AOS metabolism in the Δ*VaAly17B* mutant while not affecting extracellular alginate degradation (Fig. 2*G*). However, due to the lack of the prerequisite for the conversion of disaccharides into monomers, the Δ*VaAly17B* mutant could not maintain proper redox conditions. Therefore, these data indicated that *Va*Aly17B is essential for redox homeostasis and, thus, may be important for the biorefining of alginate.

## Discussion

Alginate is the major saccharide of the cell wall of brown seaweed, and its saccharification is a critical prerequisite for the efficient bioconversion of brown algae. In addition to converting alginate into AOSs, the complete degradation of AOSs into monomers by Oals is essential for alginate utilization. Bioinformatic analysis showed that the tandem-located Oal pair of *Va*Aly17A and *Va*Aly17B is present in all identified alginate-degrading *Vibrio* species in the CAZy database, indicating that the core pathway for the conversion of AOS into monomer in *Vibrio* species may originate from the same ancestor, which has been suggested by Hehemann *et al.* (25). The members of the Oal pair probably play similar physiological roles in AOS metabolism *in vivo*. For example, in *V. splendidus* strain 12B01, the deletion of *Va*Aly17A homolog OalB led to no growth with alginate, and when *Va*Aly17B homolog OalC was missing, cells showed a large lag to reach the stationary phase in the presence of alginate (19). These observations are in line with those in *V. alginolyticus* ATCC 17749. The different physiological functions of *Va*Aly17A and *Va*Aly17B are proven to result from their distinct biochemical characteristics: *Va*Aly17A could only degrade AOSs larger than disaccharides, whereas *Va*Aly17B is capable of converting disaccharides into monomers but cannot degrade AOSs larger than disaccharides. However, when enough unsaturated disaccharides were supplied, the Δ*VaAly17A* mutant still could not grow (data not shown). This may be caused by the significantly different activities between *Va*Aly17A and *Va*Aly17B, suggesting that *Va*Aly17A is the major actor in energy supply during AOS metabolism. Alternatively, *Va*Aly17B is mainly responsible for removing accumulated disaccharides, which is important for intracellular redox homeostasis. Therefore, our data suggested that Oal cocktails are likely required for efficient conversion of brown algae to fermentable sugar. Compared to other Oals, *Va*Aly17A and *Va*Aly17B have lower optimal temperatures (Table S2). Moreover, most identified PL17 Oals are polyM-specific enzymes, except for the bifunctional lyases of OalY1 and OalY2 from *Halomonas* sp. QY114 (43) and polyMG-specific Oals of OalB from *V*. *splendidus* 12B01 (30) and OalS17 from *Shewanella* sp. Kz7 (45). The determinants of substrate specificity remain poorly understood. Our recent study on PL15 family alginate lyases suggested that substrate preference is influenced by the distances between catalytic residues and their corresponding subsites (46). Consistent with this, structural analyses of characterized PL17 alginate lyases, which exhibited higher activity toward polyM, revealed that the distance between the catalytic base (Tyr) and the C5-H of the sugar ring at the +1 subsite, as well as the distance between the catalytic acid (Tyr) and the O4 of the glycosidic bond linking the sugars at the −1 and +1 subsites, are shorter in the presence of M-type substrates compared to G-type substrates (Table S3). In the future, the crystal structures of alginate lyases in complex with different types of oligosaccharides are required to fully elucidate the mechanism underlying substrate preference.

A close-up view of the structures of *Va*Aly17A and *Va*Aly17B revealed that one loop called Loop1 in the active groove may be the key factor resulting in different biochemical characteristics, thereby leading to different physiological functions. In *Va*Aly17A, the ends of the active groove are comparatively open, allowing trisaccharide binding. When interacting with disaccharides, the affinity of *Va*Aly17A is too low to effectively bind the substrate. The loop-swapping experiment of *Va*Aly17A and *Va*Aly17B further demonstrated the key role of Loop1 in the recognition of the minimal degrading substrate. A similar pattern is also observed in PL17 Oals containing short Loop1. For example, Alg17C from *Saccharophagus degradans* 2-40 has a minimal degrading substrate of 3 (34, 37). Oal Aly6 from *Flammeovirga* sp. strain MY04 (39) and OalV17 from *Vibrio* sp. SY01 (32) cannot degrade disaccharides. Although AlgSH17 from *Microbulbifer* sp. SH-1 exhibited activity toward disaccharides, the catalytic product analysis showed that it could only convert AOSs larger than disaccharides into disaccharides while not further degrading the produced disaccharides into monomers (40). In addition, PL17 Oals MJ-3 from *Sphingomonas* sp. MJ-3 (41) and HyAly-I from *Hydrogenopahaga* sp. strain UMI-18 (42) displayed much lower activity toward disaccharides compared to AOSs larger than disaccharides. These observations, together, suggested that PL17 Oals containing a shorter Loop1 tend to use AOSs larger than disaccharides as their preferred substrates. In *Va*Aly17B, Loop1 is long and occupies the +2 subsite. Therefore, *Va*Aly17B mainly uses disaccharides as substrates. Consistent with this, Oal17A1 from *Agarivorans* sp. B2Z047, which contains a longer Loop1, only catalyzes disaccharides but not trisaccharides or tetrasaccharides (29). The other member, Oal17A2 of the Oal pair, which shares a similar structure with *Va*Aly17A, can degrade trisaccharides or tetrasaccharides but not disaccharides. In addition, *Va*Aly17B homolog OalC from *V. splendidus* strain 12B01 displayed the highest activity toward disaccharides (30). Therefore, the size of Loop1 is a critical factor to predict the minimal substrates of different PL17 Oals.

## Conclusion

In summary, this study showed that intercellular AOS metabolism in *V. alginolyticus* ATCC 17749 is stepwise achieved by a PL17 Oal pair of *Va*Aly17A and *Va*Aly17B. This process may be universal in all alginate-degrading *Vibrio* species. *Va*Aly17A is critical for cell growth with alginate and is capable of degrading AOSs larger than disaccharides, while *Va*Aly17B is responsible for the degradation of disaccharides, which is important to maintain redox homeostasis. The loop named Loop1, located in the active groove, is a key determinant of the distinct minimal substrates for *Va*Aly17A and *Va*Aly17B. In *Va*Aly17A, the shorter Loop1 forms a relatively open groove, allowing for the binding of larger substrates. In contrast, the longer Loop1 in *Va*Aly17B occupies one end of the active site, resulting in a shorter catalytic cleft that accommodates only smaller substrates, such as disaccharides. Loop-swapping results highlight the essential role of the shorter Loop1 in facilitating effective interaction with substrates larger than disaccharides. The length of Loop1 may be a hallmark for distinguishing minimal substrates of PL17 alginate lyases. This work not only gains insight into the role of a PL17-Oal pair in AOS metabolism but also provides a new strategy to distinguish the minimal recognition patterns of PL17 alginate lyases.

## Experimental procedures

### Materials

PrimeSTAR Max DNA polymerase was purchased from TaKaRa (Tokyo, Japan). Restriction enzymes, T4 DNA ligase, and protein markers were obtained from Thermo Fisher Scientific. Kanamycin, chloramphenicol, and diaminopimelic acid were purchased from Sigma-Aldrich. All molecular kits and DNA markers were purchased from TIANGEN. Sodium alginate (purity: ≥ 98%) was purchased from Sigma-Aldrich. Its M/G ratio is 1.56 (61/39), the solubility is 10 g/L at 25 °C, and the viscosity is 4 to 12 mPa s. However, its M and G arrangement is unknown. PolyG (purity: ≥ 97%) and PolyM (purity: ≥ 97%) with DP from 30 to 41 and saturated AOSs (M6, G6, M5, G5, M4, G4, M3, M2, G3, and G2) were purchased from BZ Oligo Biotech Co., Ltd. All other chemical reagents were obtained from Sangon Biotech.

### Bacterial strains and growth conditions

Microorganisms and plasmids used in this work are listed in Table S4. *E*. *coli* strains were grown in lysogeny broth (LB) at 37 °C, and *V. alginolyticus* ATCC 17749 strains were cultured at 30 °C if not specified otherwise. For the preparation of alginate medium, a modified M9 minimal salt solution (90 mM Na_2_HPO_4_, 22 mM KH_2_PO_4_, 18.8 mM NH_4_Cl, 345 mM NaCl, and 1 mM MgSO_4_ per L) was supplemented with 0.2% sodium alginate. In glucose medium, sodium alginate was substituted equimolarly by glucose. If not specified, the sodium alginate used in this work was referred to as alginate. The optical density (OD_600 nm_) of the *V. alginolyticus* ATCC 17749 strains was measured at 600 nm in 300-mL flasks containing 100 ml of medium agitated at 200 rpm. When needed, antibiotics were used as follows: 34 μg/ml chloramphenicol or 30 μg/ml kanamycin was used for *E. coli*; 5 μg/ml chloramphenicol was used for *V. alginolyticus*. During the conjugation, 0.5% diaminopimelic acid (DAP) was added to culture the donor *E. coli* strain X7213.

### Genetic and molecular biology techniques

DNA fragment purification, digestion, ligation, and plasmid transformation were carried out using standard molecular and genetic techniques (47). DNA products were confirmed by sequencing at Beijing Tsingke Biotech Co., Ltd and analyzed using the software Vector NTI Advance 11.5.1 (Invitrogen). All DNA sequences used in this work are listed in Table S5.

### Identification and analysis of alginate-degrading genes

Using the alginate-utilizing protein sequences from *Gramella forsetii* KT0803 (48), *V*. *splendidus* 12B01 (19), and *Sphingomonas* sp. A1 (49) as queries to construct a local database, proteins possibly involved in alginate metabolism were identified by BLASTP homology searching in the genome of *V. alginolyticus* ATCC 17749 (GenBank accession numbers CP006718 and CP006719) (50). The conserved domains of Oals were predicted by SMART (https://smart.embl.de) (51). The residues coding for a putative signal peptide were analyzed by the SignalP 6.0 server (https://services.healthtech.dtu.dk/services/SignalP-6.0/) (52). The theoretical molecular weight (MW) was calculated by the ExPASy server (https://www.expasy.org/compute-pi/). The multiple sequence alignment was performed by Clustal Omega (https://www.ebi.ac.uk/Tools/msa/clustalo/) (53) and further visualized by ESPript 3.0 (https://espript.ibcp.fr/ESPript/ESPript/) (54). The phylogenetic tree was carried out by the software MEGA 11.0.13 (55), and the reference alginate lyases were selected from the CAZy database (Last update: 2024-05-24, http://www.cazy.org/) (56).

### Construction of mutant strains

A homologous recombination method was used to obtain unmarked deletions of *VaAly17A* and *VaAly17B*. First, 2000 bp-flanking fragments of *VaAly17A* and *VaAly17B* obtained by fusion PCR were ligated with KpnI/SmaI-digested pRE112 to yield plasmids pZLZ09 and pZLZ10, respectively. Then, plasmids pZLZ09 and pZLZ10 were transformed into *V. alginolyticus* ATCC 17749 by conjugation as follows: 500 μl of plasmid-containing *E. coli* X7213 culture in the exponential phase was mixed with 800 μl of *V. alginolyticus* ATCC 17749 in the exponential phase and mated overnight in LB medium supplemented with 0.5% DAP at 30 °C. Subsequently, the mixture was suspended in 10 ml of LB medium, and 100 μl was spread-plated in the LB plate in the presence of 5 μg/ml chloramphenicol for 24 h. After being confirmed by PCR, the plasmid-inserted colonies were transferred into 5 ml of fresh LB medium twice and spread-plated in the LB negative selection plate in the presence of 10% sucrose. After 30 h of incubation at 30 °C, the colonies were cultured in LB for 6 h, and PCR was performed to obtain the mutants. The mutants were designated the Δ*VaAly17A* mutant and the Δ*VaAly17B* mutant, respectively.

For genetic complementation of Δ*VaAly17A* and Δ*VaAly17B* mutants, a plasmid pBBR1MCS-2-Cm with chloramphenicol resistance was constructed as follows: The pBBR1MCS-2 was used as a backbone, and its kanamycin gene with the promoter was substituted by the chloramphenicol gene with the promoter from the plasmid pRE112 to generate the plasmid pBBR1MCS-2-Cm. The genes of *VaAly17A* and *VaAly17B* with their own promoter regions ligated with XhoI/SmaI-digested pBBR1MCS-2-Cm to obtain plasmids pZLZ14 and pZLZ15, respectively. Then, conjugation was carried out to transfer the plasmid into the mutant.

### Real-time quantitative PCR (RT-qPCR) analysis

For the RT-qPCR assay, cells were grown in alginate medium or glucose medium. After being harvested at different time points, the total RNA was extracted using the RNAprep Pure Cell/Bacteria Kit (TIANGEN) based on the manufacturer’s instructions. Then the Fastking gDNA Dispelling RT SuperMix Kit (TIANGEN) was used for genomic DNA elimination reactions and cDNA synthesis. All DNA sequences used in this study are shown in Table S5. The housekeeping gene *mreB* coding for actin protein was used as an internal control (57). All RT-qPCR reactions were conducted in a 20-μl volume with 10 μl of 2xSYBR Green *Pro Taq* HS Premix (Accurate Biology), 1 μl of cDNA template, 8.4 μl of ddH_2_O, and 0.3 μl of forward and reverse primers (10 μM). RT-qPCR was performed on qTOWER^3^ Series Real-Time Thermal Cyclers (Analytik Jena). The qTOWER^3^ system, qPCRsoft 4.1, was used to analyze the collected data. The comparative C^T^ method (2^−ΔΔCT^) was employed to obtain the relative expression data (58). Three independent experiments were performed, and all values reported were obtained from representative experiments.

### Preparation and analysis of cell membrane and cytoplasm proteins

*V. alginolyticus* ATCC 17749 cells were first grown in 200 ml of alginate medium to the stationary phase. After centrifugation at 10,000 *g* for 20 min, the supernatant was maintained as the extracellular fraction. Then, the pellet was resuspended in 30 mM Tris-HCl (pH 8.0), sonicated for 40 min, and centrifuged at 20,000 *g* for 1 h to obtain the supernatant. The supernatant was further ultracentrifuged at 320,000 *g* for 1 h, and the precipitate and supernatant were obtained as cell membrane and cytoplasm samples, respectively. The protein concentration of the samples was determined by the Bradford method, and 100 μg of protein was added to 50 μl of denaturing buffer (2.75 mM EDTA, 500 mM Tris-HCl, 6 M guanidine hydrochloride), followed by 30 μl of 1 M DTT, and incubated at 37 °C for 2 h. 50 μl of 1 M IAA (iodoacetamide) was added and left in the dark for 1 h. The samples were then ultrafiltered in Microcon YM-10 centrifuge tubes (Sigma-Aldrich) for 4 times (25,000 *g*, 15 min), and the protein concentration was determined. Trypsin (0.5 μg/μl) was added to the samples at a 1:25 ratio (w/w, trypsin/samples) and incubated overnight at 37 °C, followed by desalting using C18 Ziptip. Finally, the samples were detected using an Orbitrap Fusion Lumos Tribrid Mass Spectrometer (Thermo Fisher Scientific).

### Proteomic analysis

Precultures of *V. alginolyticus* ATCC 17749 were grown in glucose medium at 30 °C two times prior to inoculation. Then, wild-type cells were cultivated in either alginate medium or glucose medium at 30 °C for 20 h. After collecting, cells were lysed with SDT buffer (4% SDS, 1 mM DTT, 100 mM Tris-HCl, pH 7.6). Subsequently, 100 μl of 100 mM iodoacetamide (IAA) was added to block reduced cysteine residues, and the mixtures were incubated at 20 °C in darkness for 30 min. Finally, the samples were proteolyzed with 4 μg trypsin (Promega) in 40 μl of 25 mM NH_4_HCO_3_ buffer at 37 °C overnight. Liquid chromatography-tandem mass spectrometry (LC-MS/MS) analysis was carried out on a TimsTOF Pro mass spectrometer (Bruker). After the combination, protein searches against the sequence database of *V. alginolyticus* ATCC 17749 were performed using the MaxQuant 1.5.3.17 software with a peptide level of false discovery rate (FDR) set to 0.01 for identification and quantitation analysis. Abundance was obtained according to the proportion of the intensity-based absolute quantification (iBAQ) of a protein in the sum of iBAQs in the same sample, as shown by relative iBAQ (riBAQ) values. Values were obtained from three independent samples.

### Protein production and purification of alginate lyases and their mutants

By using primers listed in Table S5, the genes coding for *Va*Aly17A and *Va*Aly17B were amplified from the genomic DNA of *V. alginolyticus* ATCC 17749. After purification, the PCR products of *Va*Aly17A and *Va*Aly17B were ligated into NcoI/XhoI-digested pLYJ163 to generate plasmids pZLZ04 and pZLZ05, respectively. To obtain the mutant proteins *Va*Aly17A with a long Loop1 and *Va*Aly17B with a short Loop1, the loop swapping was carried out. The exchanged amino acid sequences and the schematic diagram of the loop swapping were shown in Figure 5*C*. The mutant expression vectors were constructed by fusion PCR using respective pZLZ04 and pZLZ05 as templates to generate the plasmids pLX01 and pLX02. The *Va*Aly17A with the long Loop1 of *Va*Aly17B was named *Va*Aly17A-L1B, and the *Va*Aly17B with the short Loop1 of *Va*Aly17A was named *Va*Aly17B-L1A. These expression vectors were transformed into *E*. *coli* BL21 (DE3), and cells were cultured at 37 °C in LB medium to an OD_600 nm_ of 0.8. Then, 0.1 mM isopropyl-β-d-thiogalactopyranoside (IPTG) was added to induce gene expression. After a 20-h incubation at 16 °C, the cells were collected by centrifugation at 4 °C at 9000 *g* for 15 min, suspended in 50 mM Tris-HCl buffer (pH 8.0, 200 mM NaCl), lysed by sonication, and collected by centrifugation at 4 °C at 9000 *g* for 20 min. Proteins with N-terminal His tags were purified using affinity chromatography (Cytiva), and PD-10 desalting columns (GE Healthcare) were used to remove imidazole. Protein concentrations were measured at an absorption of 280 nm using the NanoPhotometer N60 (IMPLEN). During the measurement, the molar extinction coefficients of 106,120 M^−1^ cm^−1^ and 109,100 M^−1^ cm^−1^ were obtained from the ExPASy server (https://web.expasy.org/protparam/) and applied for protein concentrations of *Va*Aly17A and *Va*Aly17B, respectively.

### Alginate lyase activity assay

Alginate lyase activity was determined by using the thiobarbituric acid (TBA) assay at 548 nm (30, 59). Enzyme reactions were carried out in a buffer containing 50 mM Tris-HCl (pH 8.0, 200 mM NaCl), enzymes (*Va*Aly17A, 7.4 μmol/L; *Va*Aly17B, 115 μmol/L; *Va*Aly17A-L1B, 3.7 μmol/L; *Va*Aly17B-L1A, 75 μmol/L), and 0.3% alginate substrates, including sodium alginate, polyM, polyG, and saturated AOSs (M6, G6, M5, G5, M4, G4, M3, G3, M2, and G2). The mixture was incubated at its optimal temperature and pH for 10 min, and the reaction was stopped by boiling the mixture for 10 min. After centrifugation at 11,000 *g* for 10 min at room temperature, 100 μl of the supernatant was mixed with 125 μl of 0.025 N H_5_IO_6_ in 0.125 N H_2_SO_4_ and reacted for 20 min at room temperature. Then, 250 μl of 2% sodium arsenite in 0.5 N HCl was added with shaking. After 2 min of incubation, the mixture was reacted with 1 ml of 0.3% TBA (pH 2.0) and boiled for 10 min. Enzyme activity was examined by measuring the increase in absorbance at 548 nm, resulting from the condensation of β-formylpyruvic acid and TBA (ε = 2.9 × 10^4^ M^−1^ cm^−1^). One unit (U) of alginate activity was defined as the amount of enzyme required to release 1 nmol of β-formylpyruvic acid per minute under the optimal reaction conditions. The sugar 2-deoxy-d-glucose was used as a standard to qualify the amount of α-keto acid (4-deoxy-l-*erythro*-5-hexoseulose uronic acid). The activity assays were carried out in triplicate, and values were shown as means ± standard deviations.

For the measurement of optimal reaction conditions, the dinitrosalicylic acid (DNS) method was used based on the release of reducing sugars from substrates. The highest activity was defined as 100%, and the relative enzyme activity was calculated for different conditions. First, 100 μl of enzyme solution (*Va*Aly17A, 12.3 μmol/L; *Va*Aly17B, 195 μmol/L) was added to 100 μl of 0.3% sodium alginate solution and mixed, and the reaction was performed under the optimal reaction conditions for 30 min. Then, 100 μl of DNS solution was added to the mixture and incubated at 100 °C for 10 min. The absorbance values of the reaction products at 540 nm were detected after cooling. The optimum temperature was tested by examining the activity at different temperatures (4, 10, 20, 30, 40, 50, and 60 °C) at pH 8.0 in the presence of 200 mM NaCl. The optimum pH was measured in the range of pH 3.0 to 12.0 (50 mM citrate-citric acid buffer, pH 3.0–6.4; 50 mM Tris-HCl, pH 6.8–9.0; 50 mM glycine-NaOH buffer, pH 9.5–12.0). The effect of NaCl concentration on enzyme activity was examined under NaCl conditions from 0 to 2.0 M (0, 0.1, 0.2, 0.4, 0.6, 0.8, 1.0, 1.5, and 2.0 M). Values reported were averaged using three independent samples.

### Enzyme kinetics assay

The kinetic parameters (*K*_m_, *V*_max_, and *k*_cat_) of *Va*Aly17A and *Va*Aly17B were determined under standard assay conditions. Reactions were carried out in 100 μl volumes containing 40 μl of appropriately diluted enzyme solution (*Va*Aly17A, 3.9 μmol/L; *Va*Aly17B, 118 μmol/L) and 60 μl of substrate at various concentrations (0.01, 0.05, 0.10, 0.30, 1.00, 3.00 mg/ml) in 50 mM Tris-HCl (pH 8.0, 200 mM NaCl). Substrates included sodium alginate, polyM, and polyG. The reaction mixtures were incubated at the optimal temperature of the enzyme for 5 min. The enzymatic activity was determined by using the thiobarbituric acid (TBA) assay at 548 nm. All assays were performed in triplicate. Initial reaction rates at each substrate concentration were calculated and plotted using GraphPad Prism software (version 9.5.0). The kinetic parameters *K*_m_ and *V*_max_ were determined by nonlinear regression fitting to the Michaelis-Menten equation. The turnover number (*k*_cat_) was calculated by dividing *V*_max_ by the concentration of the enzyme used in the reaction.

### Analysis of degradation products

Degradation products of *Va*Aly17A and *Va*Aly17B toward different AOSs were preliminarily observed by TLC with silica gel 60 plates (Merck, Darmstadt, Germany) (60). The purified enzyme (*Va*Aly17A, 7.4 μmol/L; *Va*Aly17B, 115 μmol/L) was added to 1% (w/v) AOSs in 50 mM Tris-HCl (pH 8.0, 200 mM NaCl), followed by incubation at the optimal temperature for 24 h. The reaction mixture was boiled at 100 °C for 10 min to inactivate the enzyme and subsequently centrifuged at 9000 *g* for 10 min at 4 °C. The 2-μl reaction mixture was spotted on 60 silica gel plates, which was resolved in the solvents containing *n*-butanol, acetic acid, and water [2:1:1 (v/v/v)] at room temperature for 2 h, visualized in 10% H_2_SO_4_, and heated at 100 °C for 10 min to detect sugars.

To further identify the molecular weight of oligosaccharides, the oligosaccharide solutions were detected by HPLC and ESI-MS (33). First, the enzyme reaction was carried out in 50 mM Tris-HCl (pH 8.0, 200 mM NaCl) and at the optimal temperature (30 °C for *Va*Aly17A and 20 °C for *Va*Aly17B) for different time intervals. The substrate concentration was 10 mg/ml, and the enzyme concentrations of *Va*Aly17A and *Va*Aly17B were 7.4 μmol/L and 115 μmol/L, respectively. Then, the reaction mixture was boiled for 10 min, filtered through 0.22-μm filters, and centrifuged at 9000 *g* for 15 min. A Superdex peptide 10/300 Gl gel filtration column (GE Healthcare) was used to examine the supernatant products at a flow rate of 0.3 ml/min. The elution was monitored by measuring the absorption at 235 nm. On the other hand, the reaction samples were desalted on a C-18 Ziptip (Millipore) and suspended in 10 μl of ultrapure water. ESI-MS was performed to detect the AOS products in negative ionization mode with a mass range from 0 to 2000 m/z.

### Molecular docking analysis

The protein structures based on the sequences of *Va*Aly17A and *Va*Aly17B were predicted by AlphaFold2 (61). The open-source code for AlphaFold2 was obtained from Github (https://github.com/deepmind/alphafold) and ran on a high performance computing cloud platform at Shandong University (http://cloud.sdu.edu.cn/portal/view/main.htmL). The evaluation pLDDT scores were used as criteria to select the structure. The complexes of *Va*Aly17A-trisaccharide (MMG) and *Va*Aly17B-disaccharide (MM) were constructed with Autodock Vina (62). The substrate MMG was obtained from the complex of Alg17C (PDB: 4OJZ) (37), and MM was obtained from the complex of AlyA3 (PDB: 7BM6) (35). The geometrical matching quality and interacting energy were used as criteria to select the complex. Molecular interaction was analyzed on PyMOL Version 2.5.5, and a cutoff of 4 Å was set for the selection of amino acid residues showing interaction with the substrate.

### NAD^+^/NADH ratio assay

Strains were cultured in M9 high salt medium supplemented with 0.2% casamino acid as a control to avoid the effects of other chemicals in the medium, and the cells were harvested when grown to the exponential phase. Each sample containing about 10^6^ cells was suspended in 100 μl of phosphate-buffered saline (PBS) buffer and analyzed by using an NAD^+^/NADH-Glo assay kit (Promega) following the manufacturer’s instructions (63). Luminescence was recorded using an EnSpire 2300 multimode microplate reader (PerkinElmer).

## Data availability

All the data mentioned are included in the manuscript and supporting information.

## Supporting information

This article contains supporting information (17, 29, 30, 35, 36, 37, 38, 39, 40, 41, 43, 45, 50, 66, 67, 68, 69, 70, 71, 72, 73, 74, 75, 76, 77, 78).

## Conflicts of interest

The authors declare that they have no conflicts of interest with the contents of this article.
