# Supplementary material for: A key loop in the catalytic pocket of the PL17 family of alginate lyases determines minimal substrate recognition
Source: J Biol Chem. 2025 Jul 9;301(8):110467. doi: 10.1016/j.jbc.2025.110467 (PMC12336832; doi:10.1016/j.jbc.2025.110467)
Supplement: Supporting Figures and Tables [file mmc1.docx]

Supporting Information

A key loop in the catalytic pocket of the PL17 family of alginate lyases determines minimal substrate recognition

Running title: A loop determines the PL17 substrate recognition

Xue Li^a^, Lanzeng Zhang^a^, Yongqi Tang^a^, Yi Li^a^, Xinyu Wu ^a^, Yingjie Li^a, *^, Lushan Wang^a^

^a^State Key Laboratory of Microbial Technology, Shandong University, Qingdao, 266237, China

*Address correspondence to Yingjie Li, [yingjie.li@sdu.edu.cn](mailto:yingjie.li@sdu.edu.cn)

**
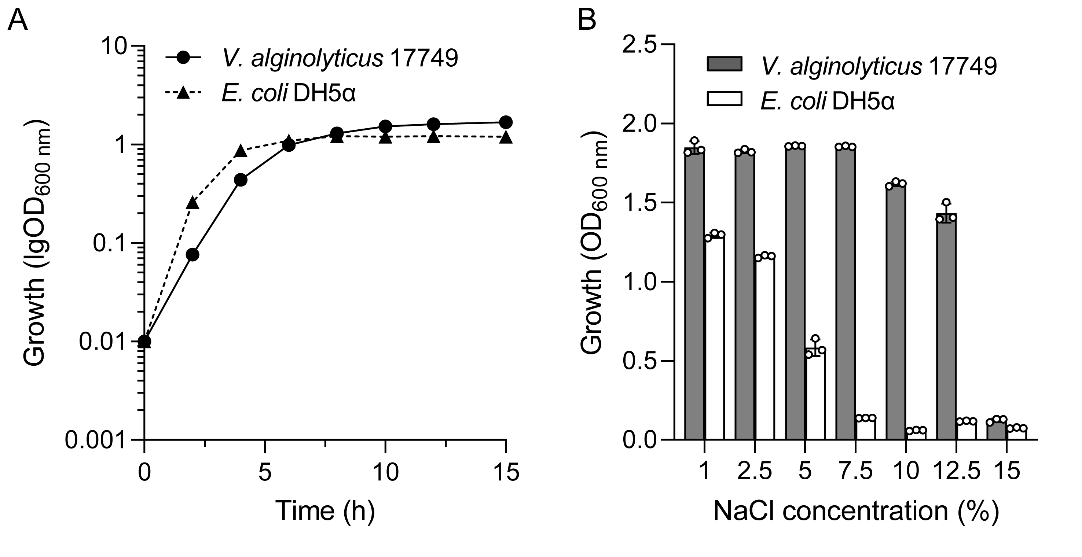
**

**Figure S1. Growth comparison of** ***V. alginolyticus* ATCC 17749 and** ***E. coli* DH5α in LB medium.** *A*, Growth curves of *V. alginolyticus* ATCC 17749 at 30℃ and *E. coli* DH5α at 37℃ in LB medium. *B*, Growth of *V. alginolyticus* ATCC 17749 and *E. coli* DH5α in LB medium containing different NaCl concentrations (1%–15%) to evaluate salt tolerance. The strains were cultured at 30°C for 24 h.

**
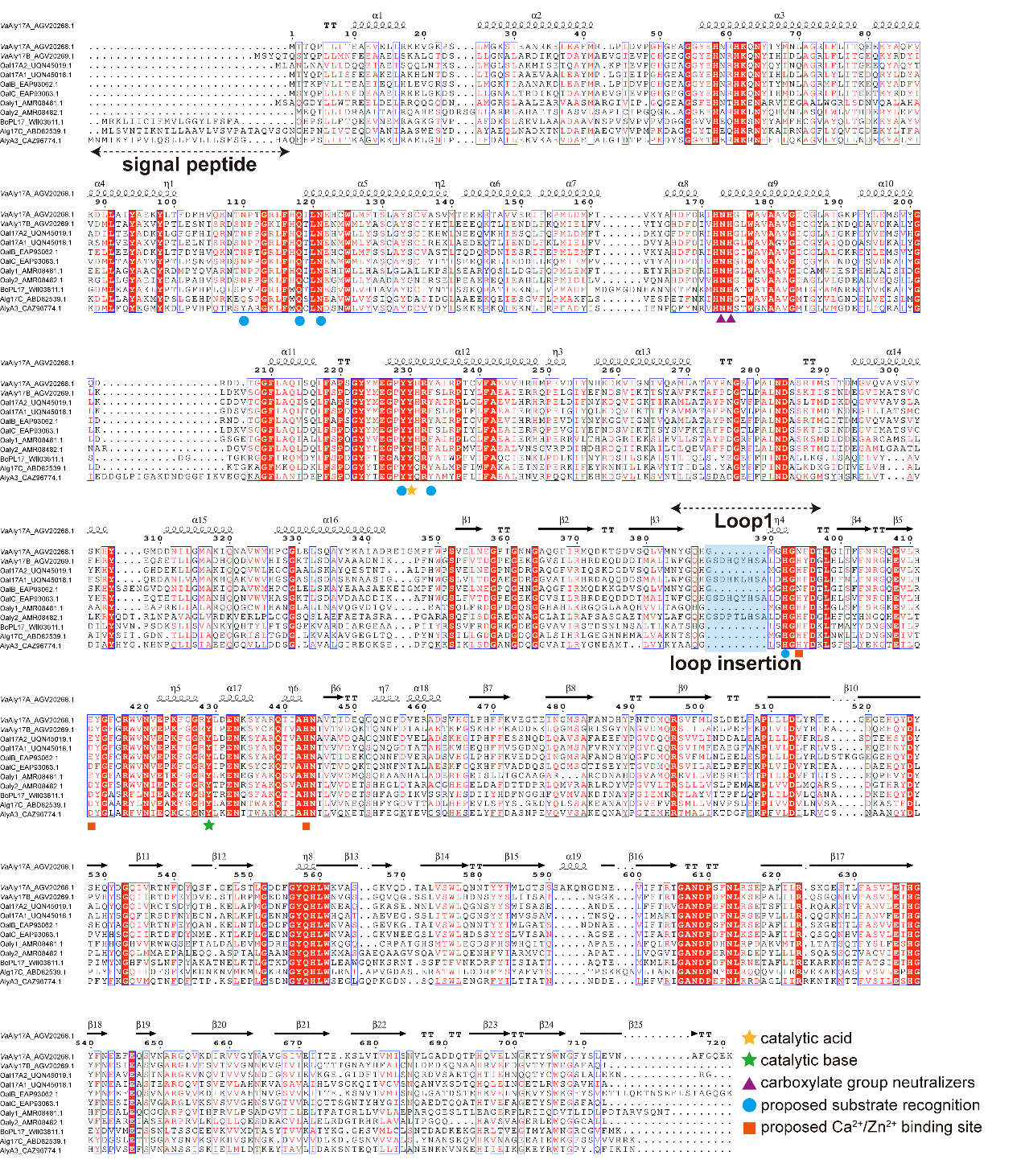
**

**Figure S2. Multiple sequence alignment of *Va*Aly17A and *Va*Aly17B.** The secondary structure elements of *Va*Aly17A are shown, with α-helices as coils, β-strands as solid arrows, and T-turns labeled as TT. Fully conserved residues are marked with a red background. Compared to *Va*Aly17A, the Loop1 of *Va*Aly17B contains an additional amino acid sequence insertion, highlighted with a blue background. Residues involved in catalysis, charge stabilization, metal-ion binding, and substrate recognition are marked using colored symbols.


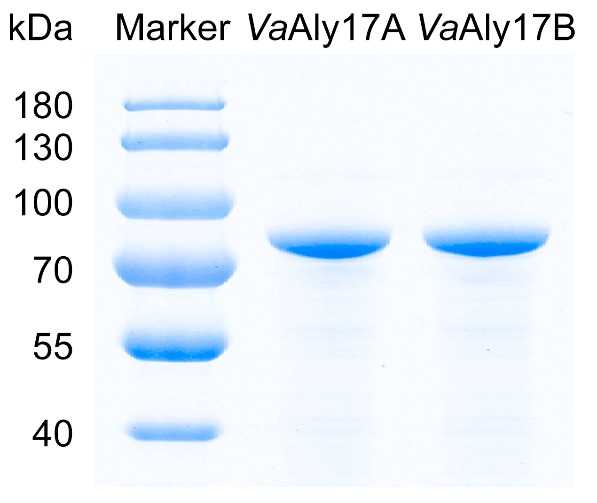


**Figure S3. SDS-PAGE analysis of the active, purified enzymes.** The molecular weights of *Va*Aly17A and *Va*Aly17B are about 80 kDa, which are consistent with their respective theoretical molecular weights of 81,525 Da and 81,023 Da.

**
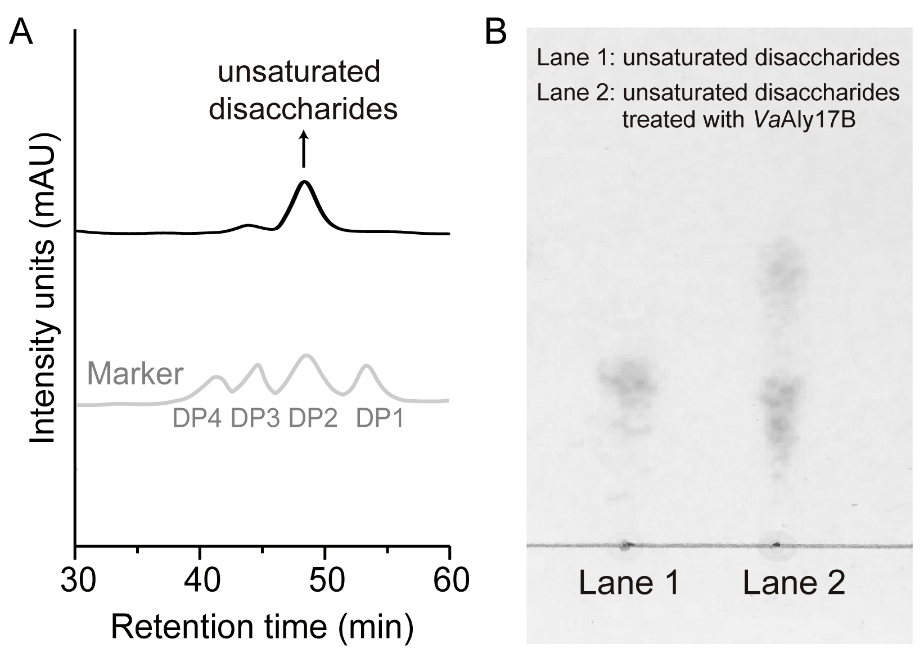
**

**Figure S4. Degradation of unsaturated disaccharides by *Va*Aly17B.** *A*, Unsaturated disaccharides were produced by a PL7 family alginate lyase, *Va*Aly7A, through its reaction with sodium alginate and were subsequently purified using fast protein liquid chromatography (FPLC). *B*, TLC analysis of the degradation of purified unsaturated disaccharides by *Va*Aly17B. Upon incubation at 20℃ for 6 hours, the unsaturated disaccharides were partially converted into monomers.


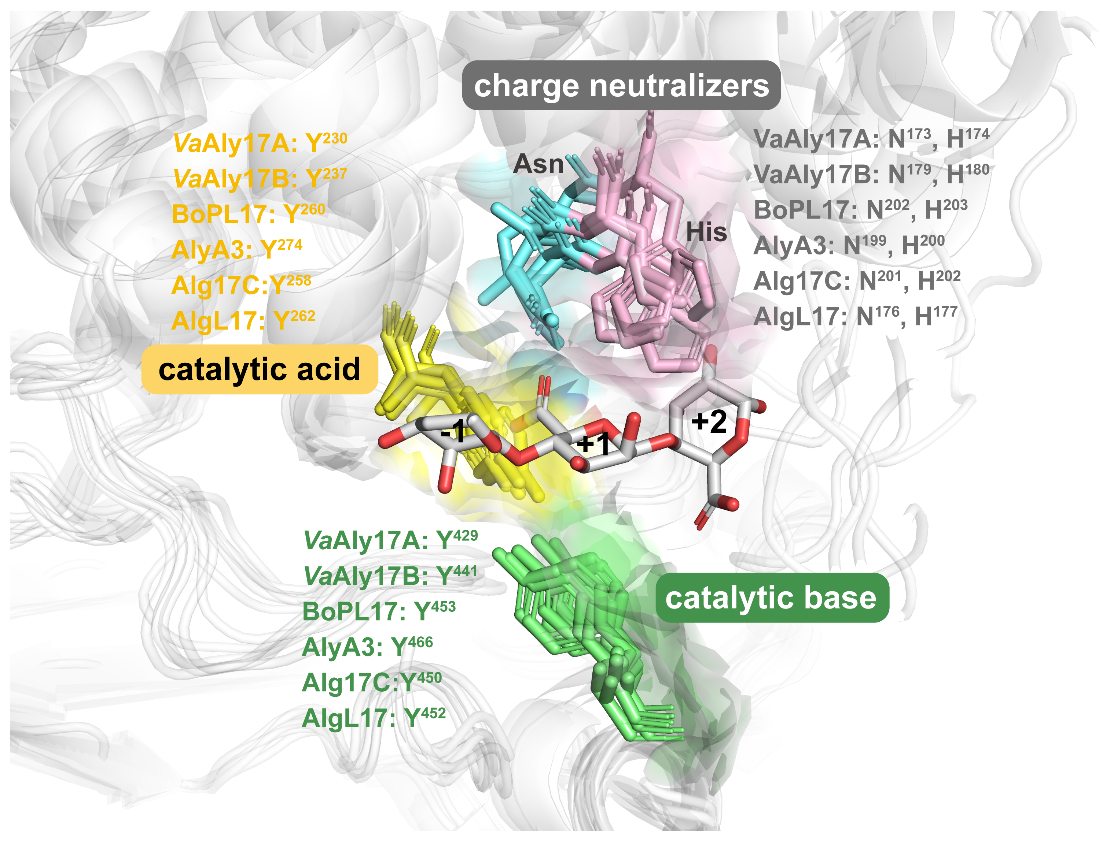


**Figure S5. Structural comparison of catalytic residues in the PL17 family alginate lyases.** Structural alignment of *Va*Aly17A, *Va*Aly17B, BoPL17 (PDB: 8BDD) (36), AlyA3 (PDB: 7BJT) (35), Alg17C (PDB: 4NEI) (37), and AlgL17 (PDB: 9IRQ) (38) indicates that the catalytic acid, catalytic base, and charge-neutralizing residues are conserved among the PL17 family alginate lyases.


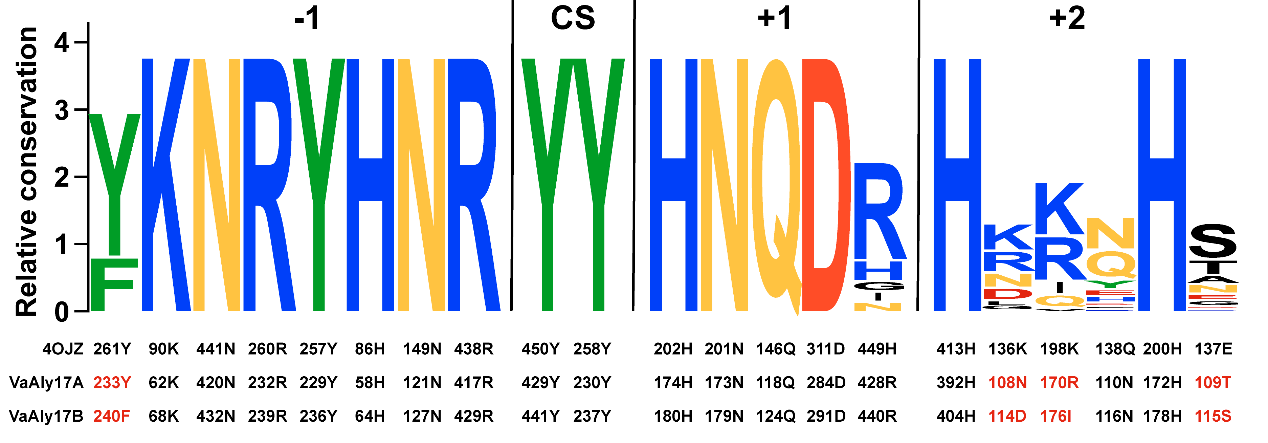


**Figure S6. The sequence profile of the PL17 family was obtained by using all candidate PL17 Oals in the CAZy database.** The ordinate indicates the relative conservation degree, and the abscissa indicates the template structure (Alg17C, PDB: 4OJZ) (37), including the amino acid type and residue number. The different residues between *Va*Aly17A and *Va*Aly17B are shown in red. Each type of amino acid is shown by abbreviated letters with a corresponding color (KRH, blue; DE, red; NQ, orange; WFY, green; others, black), and the same color suggests similar physicochemical properties. CS indicates the cleavage site. Residues within 5 Å around the active site are shown as the composition of the active site architecture.


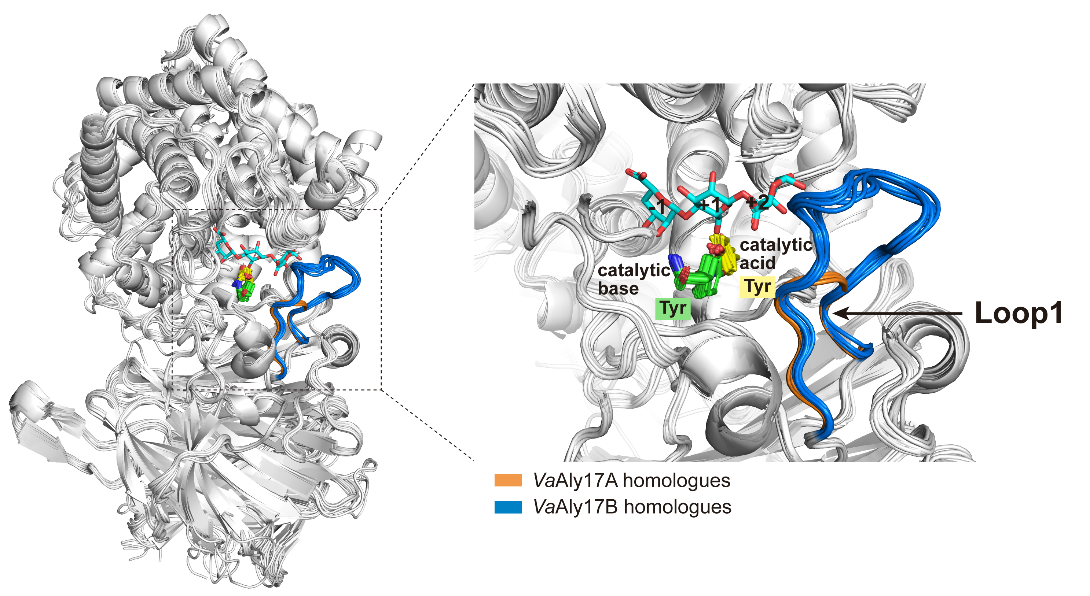


**Figure S7. Structure alignment of PL17 Oal pair in alginate-degrading *Vibrio* species.** Loop1, located around the active center, is longer in *Va*Aly17B and its homologues (blue) compared to *Va*Aly17A and its homologues (orange). The trisaccharide substrate (MMG), derived from the Alg17C substrate complex (PDB: 4OJZ) (37), is positioned at the -1 to +2 subsites of *Va*Aly17A homologues and *Va*Aly17B homologues.

**
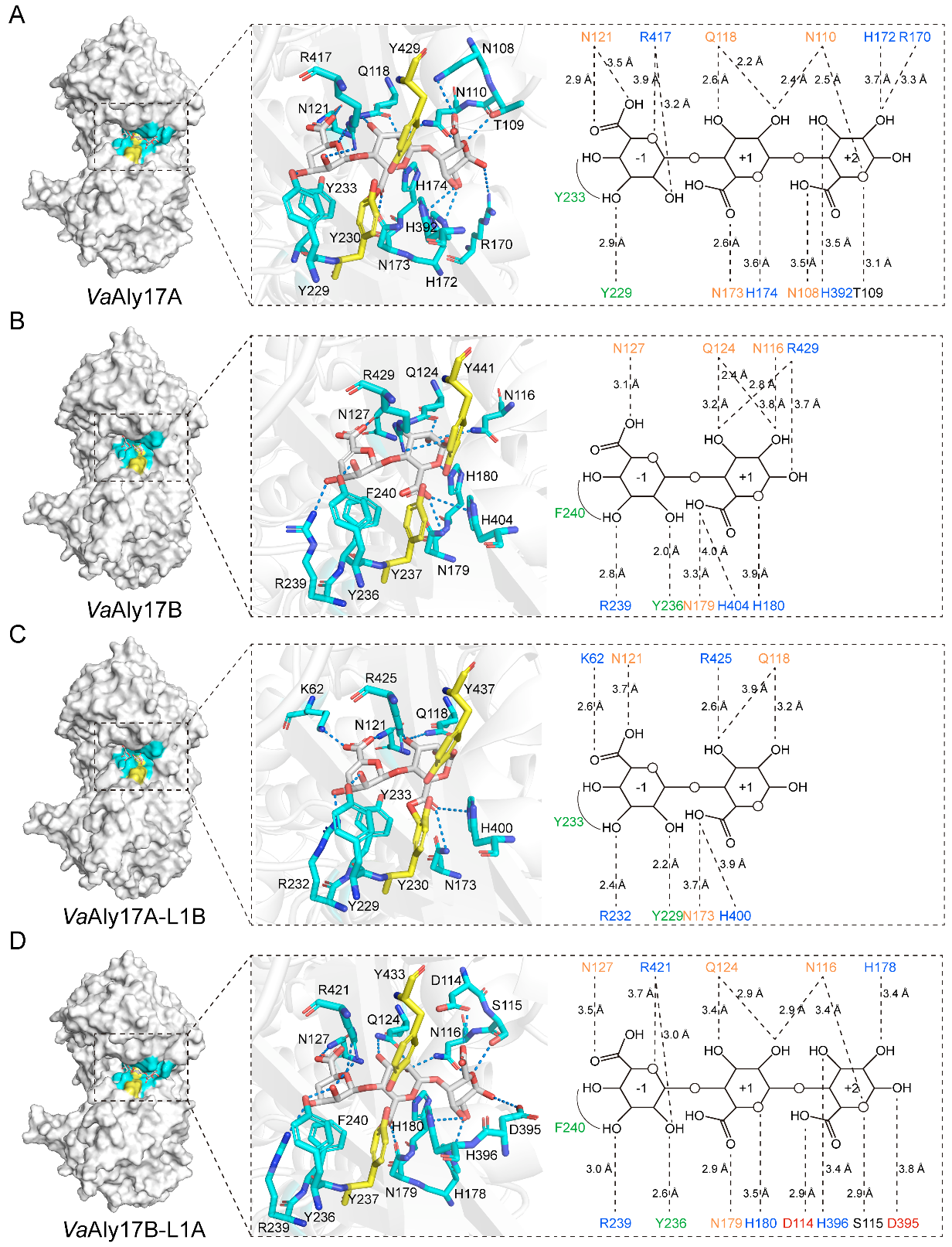
**

**Figure S8. Interaction analysis between wild-type and loop-exchanged proteins with trisaccharide and disaccharide substrates.** *A*, Molecular docking analysis of the *Va*Aly17A-MMG complex. The substrate of trisaccharide (MMG) was obtained from the complex of Alg17C (PDB: 4OJZ) (37). A close-up view of the active site is shown in the middle, and the interaction between residues and the ligand was analyzed. *B*, Molecular docking analysis of the *Va*Aly17B-MM complex. The substrate of disaccharide (MM) was obtained from the complex of Alya3 (PDB: 7BM6) (35). Compared to *Va*Aly17A, the right end of the active groove in *Va*Aly17B is too close to allow the binding of AOS larger than disaccharide. (*C*) and (*D*) Molecular docking analyses of *Va*Aly17A-L1B-MM and *Va*Aly17B-L1A-MMG. The structures of the mutant proteins *Va*Aly17A-L1B and *Va*Aly17B-L1A were predicted by AlphaFold2. The amino acid residues interacting with the ligand are shown in cyan. The residues for the β-elimination reaction are shown in yellow.


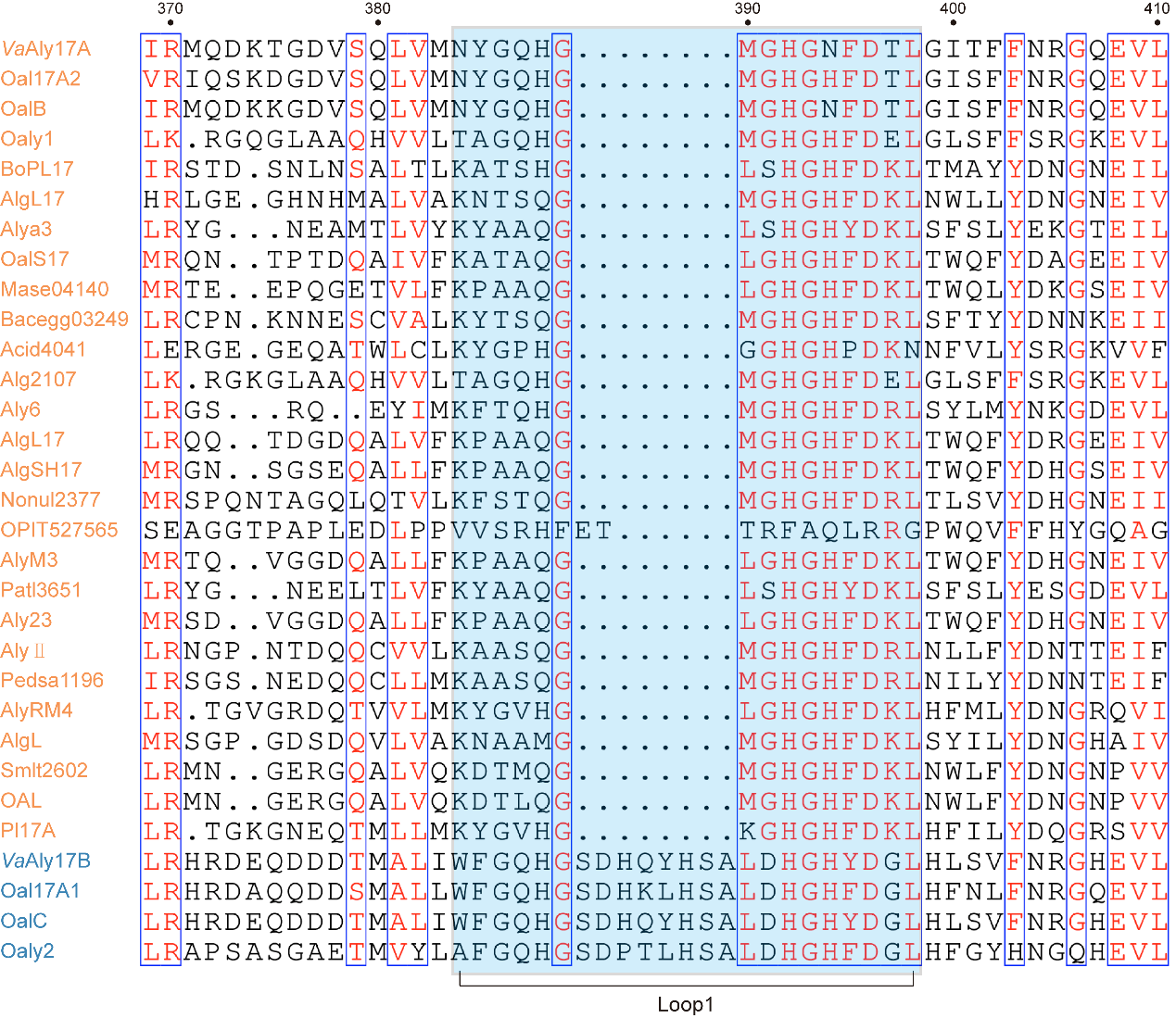
**Figure S9. Sequence alignment of Loop1 in all characterized PL17 Oals.** The Loop1 was indicated in a blue box, two types of Loop1 were observed. *Va*Aly17A, *Va*Aly17B, and other characterized PL17 Oals were used, including Oal17A2 (UQN45019.1) from *Agarivorans* sp. B2Z04 (29); Oal17A1 (UQN45018.1) from *Agarivorans* sp. B2Z047 (29); Mase04140 (AFS36377.1) from *Alteromonas macleodii* ATCC 27126 (66); Bacegg03249 (EEC52271.1) from *Bacteroides eggerthii* DSM 20697 (66); BoPL17 (WII03611.1) (PDB: 8BDD) from *Bacteroides ovatus* CP926 (36); Acid4041 (ABJ85006.1) from *Candidatus Solibacter usitatus* Ellin6076 (66); Alg2107 (QQK64493.1) from *Cobetia* sp. cqz5-12 (67); Aly6 (ANQ49918.2) from *Flammeovirga* sp. MY04 (39); Oaly2 (AMR08482.1) from *Halomonas* sp. QY114 (43); Oaly1 (AMR08481.1) from *Halomonas* sp. QY114 (43); AlgL17 (ATG71374.1) (PDB: 9IRQ) from *Microbulbifer* sp. ALW1 (38); AlgSH17 (QIL90639.1) from *Microbulbifer* sp. SH-1 (40); Nonul2377 (WP_036584315.1) from *Nonlabens ulvanivorans* (66); AlyM3 (MBZ2193586.1) from *Pseudoalteromonas arctica* M9 (68); Patl3651 (ABG42153.1) from *Pseudoalteromonas atlantica* T6c (66); Aly23 (QWT68652.1) from *Pseudoalteromonas carrageenovora* ASY5 (69); AlyⅡ (BAA19848.1) from *Pseudomonas* sp. OS-ALG-9 (70); Pedsa1196 (ADY51764.1) from *Pseudopedobacter saltans* DSM 12145 (66); AlyRM4 (UVF39826.1) from *Rhodothermus marinus* MAT378 (71); Alg17C (ABD82539.1) (PDB: 4NEI) from *Saccharophagus degradans* 2-40 (37); OalS17 (AHW45238.1) from *Shewanella* sp. Kz7 (45); AlgL (AEM45874.1) from *Sphingomonas* sp. MJ-3 (41); Smlt2602 (CAQ46078.1) from *Stenotrophomonas maltophilia* K279a (72); OAL (AGM38186.2) from *Stenotrophomonas maltophilia* KJ-2/KCTC32000 (73); Pl17A (QMS47797.1) (72); OalB (EAP93062.1) from *Vibrio splendidus* 12B01 (30); OalC (EAP93063.1) from *V. splendidus* 12B01 (30); AlyA3 (CAZ96774.1) (PDB: 7BJT) from *Zobellia galactanivorans Dsij*^T^ (35). Protein structures used for alignment were predicted with AlphaFold2.

**Table S1 Substrate speciﬁcities of *Va*Aly17A-L1B and *Va*Aly17B-L1A toward different AOSs**

|  | Mean specific activity (U/mg) ± SD | |
| --- | --- | --- |
| Substrate | *Va*Aly17A-L1B | *Va*Aly17B-L1A |
| Dimer-M2 | 0.15 ± 0.05 | 0.25 ± 0.01 |
| Dimer-G2 | 0.05 ± 0.01 | 0.15 ± 0.01 |
| Trimer-M3 | 0.41 ± 0.13 | 3.38 ± 0.03 |
| Trimer-G3 | 0.11 ± 0.04 | 0.25 ± 0.01 |
| Tetramer-M4 | 0.45 ± 0.08 | 1.95 ± 0.02 |
| Tetramer-G4 | 0.12 ± 0.05 | 0.53 ± 0.05 |
| Pentamer-M5 | 9.07 ± 0.73 | 4.20 ± 0.09 |
| Pentamer-G5 | 5.76 ± 0.29 | 2.39 ± 0.12 |
| Hexamer-M6 | 13.48 ± 0.52 | 4.83 ± 0.07 |
| Hexamer-G6 | 7.79 ± 0.60 | 3.73 ± 0.12 |

**Table S2 Characterized PL17 family alginate lyases from different sources**

| **Name** | **Optimal condition** | | **Action mode** | **Specific activity** | **Substrate polymerization degree (DP)** | **Source** | **Reference** |
| --- | --- | --- | --- | --- | --- | --- | --- |
|  | **Temp (℃)** | **pH** |  |  |  |  |  |
| *Va*Aly17A | 30 | 7.5–8.0 | exo | alginate (114 U/mg) > polyM (95 U/mg) > polyG (83 U/mg) | ≥3 | *V. alginolyticus* ATCC 17749 | This study |
| *Va*Aly17B | 20 | 7.5 | exo | alginate (3.2 U/mg) > polyM (1.8 U/mg) > polyG (1.1 U/mg) | 2 | *V. alginolyticus* ATCC 17749 | This study |
| AlyA3 | 40 | 7.0 | exo | polyM > polyMG | ≥3 | *Z. galactanivorans* Dsij^T^ | (35) |
| OalB | 30 | 7.0 | exo | polyMG (79 U/mg) > polyM (46 U/mg) > polyG (33 U/mg) > sodium alginate (20 U/mg) | ≥2 | *V. splendidus* 12B01 | (30) |
| OalC | 35 | 7.5 | exo | polyM (76 U/mg) > polyMG (28 U/mg) > sodium alginate (21 U/mg) > polyG (3 U/mg) | 2 | *V. splendidus* 12B01 | (30) |
| AMOR_PL17A | 70 | 5.0–6.0 | exo | alginate (0.11 g/L) > polyM (0.095 g/L) > polyG (0.05 g/L) | - | Uncultured bacterium | (72) |
| OAL | 37 | 7.5 | endo | polyM (120%) > alginate (100%) > polyMG (60%) > polyG (20%) | - | *S. maltophilia* KJ-2 | (73) |
| AlgL | 50 | 6.5 | endo | polyM (170%) > polyMG (115%) > alginate (100%) > polyG (80%) | - | *Sphingomonas* sp. MJ-3 | (41) |
| OalS17 | 50 | 6.2 | - | alginate (32 U/mg) > polyM (24 U/mg) > polyG (5 U/mg) | - | *Shewanella* sp. Kz7 | (45) |
| Alg17C | 40 | 6.0 | exo | - | - | *S. degradans* 2-40 | (37) |
| AlyRm4 | 81 | 6.5 | exo | - | - | *R. marinus* MAT378 | (71) |
| Aly23 | 35 | 6.0 | exo + endo | polyM (169%) > alginate (100%) > polyG (16%) | - | *P. carrageenovora* ASY5 | (69) |
| AlgSH17 | 30 | 7.0 | exo + endo | polyM (117 U/mg) > alginate (56 U/mg) > polyG (30 U/mg) | ≥2 | *Microbulbifer* sp. SH-1 | (40) |
| AlgL17 | 35 | 8.0 | exo | polyM (100%) > alginate (50%) > polyG (10%) | - | *Microbulbifer* sp. ALW1 | (38) |
| OalY1 | 45 | 7.05 | exo | alginate (100%) > polyG (92.5%) > polyM (89.4%) | - | *Halomonas* sp. QY114 | (43) |
| OalY2 | 50 | 6.6 | exo | alginate (100%) > polyG (91.6%) > polyM (90.8%) | - | *Halomonas* sp. QY114 | (43) |
| Aly6 | 40 | 7.0 | exo | alginate (726 U/mg) > polyM (525 U/mg) > polyG (196 U/mg) | ≥3 | *Flammeovirga* sp. MY04 | (39) |
| BoPL17 | - | 6.5 | exo | polyM | - | *B. ovatus* CP926 | (36) |
| OalC17 | 45 | 7.8 | exo | polyM (68 U/mg) > sodium alginate (37 U/mg) > polyG (20 U/mg) | - | *Cellulophaga* sp. SY116 | (74) |
| Oal17A1 | - | - | - | - | 2 | *Agarivorans* sp. B2Z047 | (29) |
| Oal17A2 | - | - | - | - | ≥3 | *Agarivorans* sp. B2Z047 | (29) |

**Table S3 Spatial distances between catalytic residues of the PL17 family Oals and their substrates**

| Name | Base | Dist. (Å) | Acid | Dist. (Å) | Substrate preference | References |
| --- | --- | --- | --- | --- | --- | --- |
| *Va*Aly17A | Tyr^429^ | **M 3.9** | Tyr^230^ | **M 3.3** | polyM | This study |
|  |  | G 5.6 |  | G 3.5 |  |  |
| *Va*Aly17B | Tyr^441^ | **M 4.1** | Tyr^237^ | **M 3.6** | polyM | This study |
|  |  | G 5.7 |  | G 3.8 |  |  |
| AlyA3 | Tyr^466^ | **M 4.7** | Tyr^274^ | **M 2.9** | polyM | (35) |
|  |  | G 6.4 |  | G 3.1 |  |  |
| BoPL17 | Tyr^453^ | **M 5.2** | Tyr^260^ | **M 3.2** | polyM | (36) |
|  |  | G 6.9 |  | G 3.3 |  |  |
| AlgL17 | Tyr^424^ | **M 4.5** | Tyr^233^ | **M 2.9** | polyM | (38) |
|  |  | G 6.2 |  | G 3.1 |  |  |

**Table S4 Strains and plasmids used in this study**

| **Strain or plasmid** | **Description** | **Source or reference** |
| --- | --- | --- |
| **Strains** |  |  |
| *Vibrio alginolyticus* ATCC 17749 | Wild-type | (50) |
| *E. coli* DH5α | The host strain used for general cloning | DINGGUO CHANGSHENG (Beijing, China) |
| *E. coli* BL21(DE3) | The host strain used for recombinant protein production | DINGGUO CHANGSHENG (Beijing, China) |
| *E. coli* CC118 | The strain used for the construction of deletion plasmids | (75) |
| *E. coli* X7213 | The donor strain for conjugation | (76) |
| Δ*VaAly17A* | *VaAly17A* deletion mutant in strain *V. alginolyticus* ATCC 17749 | This study |
| Δ*VaAly17B* | *VaAly17B* deletion mutant in strain *V. alginolyticus* ATCC 17749 | This study |
| Δ*VaAly17A*+pZLZ14 | *VaAly17A* complementation strain | This study |
| Δ*VaAly17B*+pZLZ15 | *VaAly17B* complementation strain | This study |
| **Plasmids** |  |  |
| pLYJ163 | Km^r^, vector for the expression of proteins in *E. coli* | Laboratory storage |
| pRE112 | *oriT*, *oriV*, *sacB*, Cm^r^, counterselectable suicide plasmid | (77) |
| pBBR1MCS-2 | Km^r^, mobilizable broad-host-range vector | (78) |
| pBBR1MCS-2-Cm | Cm^r^, mobilizable broad-host-range vector | This study |
| pZLZ04 | pLYJ163 plus *VaAly17A* | This study |
| pZLZ05 | pLYJ163 plus *VaAly17B* | This study |
| pLX01 | pLYJ163 plus *VaAly17A* with the long loop1 of *VaAly17B* | This study |
| pLX02 | pLYJ163 plus *VaAly17B* with the short loop1 of *VaAly17A* | This study |
| pZLZ09 | pRE112 plus 2-kb fused flanking section of *VaAly17A* | This study |
| pZLZ10 | pRE112 plus 2-kb fused flanking section of *VaAly17B* | This study |
| pZLZ14 | pBBR1MCS-2-Cm plus *VaAly17A* with its own promoter | This study |
| pZLZ15 | pBBR1MCS-2-Cm plus *VaAly17B* with its own promoter | This study |

**Table S5 Primers used in this study**

| **Primers** | **Sequence (5'-3')** |
| --- | --- |
| **Construction of Expression Plasmids** | |
| VaAly17A-F | CCGCTCGAGTTATTTCTCCTGCCCGAATG |
| VaAly17A-R | CATGCCATGGCGCCTCTAGACGTACCAGGT |
| VaAly17B-F | CCGCTCGAGTTAAATTTGTGCGAATGCGC |
| VaAly17B-R | CATGCCATGGCGCTAGCGGGTCGCCTATTC |
| VaAly17A-longer L1-F | ATGGTCTAGCGCTGAGTGGTATTGGTGATCGCTGCCGTGTTGACCGTAGTTCATCAC |
| VaAly17A-longer L1-R | GGCAGCGATCACCAATACCACTCAGCGCTAGACCATGGTAACTTCGATACGCTTGGC |
| VaAly17B-shorter L1-F | CCATCGTAGTGACCGTGGTCTAGGCCGTGTTGACCAAACCAGATAAG |
| VaAly17B-shorter L1-R | GTTTGGTCAACACGGCCTAGACCACGGTCACTACGATGGTCTGCACCTGAG |
| **Construction of Knockout Plasmids** | |
| *VaAly17A*-F1 | GGGGTACCTGGAAGTAACGCTCGCCAGT |
| *VaAly17A*-R1 | CTGGGTTTGGTAGCTCATTATTCTTTACCACCCTCAGATG |
| *VaAly17A*-F2 | CATCTGAGGGTGGTAAAGAATAATGAGCTACCAAACCCAG |
| *VaAly17A*-R2 | TCCCCCGGGCATGATTACGCCTTCATCGT |
| *VaAly17B*-F1 | CCGCTCGAGACGGAAAGAACCAGACTAGT |
| *VaAly17B*-R1 | ACATATAATAAGGTTTGAACTTCTTTACCACCCTCAGATG |
| *VaAly17B*-F2 | CATCTGAGGGTGGTAAAGAAGTTCAAACCTTATTATATGT |
| *VaAly17B*-R2 | TCCCCCGGGGCTATTAGTATTCGGCTGTTAT |
| **Construction of Complementation Plasmids** | |
| *VaAly17A*-F | TCCCCCGGGACCATGCTTTATTAAAAGGTC |
| *VaAly17A*-R | CCGCTCGAGTTATTTCTCCTGCCCGAATG |
| *VaAly17B*-F | TCCCCCGGGTAAAAGTGAAACGCGAAGCGAAAGG |
| *VaAly17B*-R | CCGCTCGAGTTAAATTTGTGCGAATGCGCCTTCC |
| **RT-qPCR** |  |
| *mreB*-qF | CGATCTATCTATCGATTTAGGTAC |
| *mreB*-qR | TGACGAATAGCAACTACAGA |
| *VaAly17A*-qF | CGATTTTGTTGACTGAAGCA |
| *VaAly17A*-qR | TCTAGAGGCAGACGCATGAA |
| *VaAly17B*-qF | GCTACCAAACCCAGTCTTAC |
| *VaAly17B*-qR | TAGTGCATTTCCTAATAGGC |
| *VaAly7A*-qF | AGAAGTTCGTGAGCGAGAAA |
| *VaAly7A*-qR | TAGCCATTGCAAGGTTAATC |
| *VaAly7B*-qF | ATCATGACTACTCGCCGTTA |
| *VaAly7B*-qR | CTGTAATAGTACGGCGACCA |
| *VaAly7C*-qF | ATCGGAAGGAATAGTGATCT |
| *VaAly7C*-qR | TTACCAACTCTTGCTATCGC |
| *kdgM*-qF | TTGATACGTTGGGCATAAGT |
| *kdgM*-qR | TATTCGTTGCTTCCACATTA |
| *toaA*-qF | CCATCCACCATAACATCTTA |
| *toaA*-qR | TTGCCTACTTCTTCTTCTTA |
| *kdgF*-qF | CCAATCGCGCCTTTGTCGAA |
| *kdgF*-qR | TGAGAACCCGTGGGAAGAGCTAGGC |
| *kdgK*-qF | CTTTGGTCAAACGAGATAAA |
| *kdgK*-qR | AATCATTAAACATCGCGGTC |
| *dehR*-qF | GACTAAACCTATCATTGGTT |
| *dehR*-qR | CATCTTTATTGAGGTCCATT |
| *kdgA*-qF | ACGTGATTTCTGCGCAAGGC |
| *kdgA*-qR | GCAAACCTAAAAGTAATTCCTG |

**References**

17. Arntzen, M., Pedersen, B., Klau, L., Stokke, R., Oftebro, M., Antonsen, S., Fredriksen, L., Sletta, H., Aarstad, O., Aachmann, F., Horn, S., and Eijsinka, V. (2021) Alginate degradation: insights obtained through characterization of a thermophilic exolytic alginate lyase. *Appl. Environ. Microbiol.* **87**, e0239920

29. Sun, X. K., Gong, Y., Shang, D. D., Liu, B. T., Du, Z. J., and Chen, G. J. (2022) Degradation of alginate by a newly isolated marine bacterium *agarivorans* sp. B2Z047. *Mar. Drugs* **20,** 254

30. Jagtap, S. S., Hehemann, J. H., Polz, M. F., Lee, J. K., and Zhao, H. M. (2014) Comparative biochemical characterization of three exolytic oligoalginate lyases from *Vibrio splendidus* reveals complementary substrate scope, temperature, and pH adaptations. *Appl. Environ. Microbiol.* **80**, 4207–4214

35. Jouanneau, D., Klau, L. J., Larocque, R., Jaffrennou, A., Duval, G., Le Duff, N., Roret, T., Jeudy, A., Aachmann, F. L., Czjzek, M., and Thomas, F. (2021) Structure-function analysis of a new PL17 oligoalginate lyase from the marine bacterium *Zobellia galactanivorans* Dsij^T^. *Glycobiology* **31**, 1364–1377

36. Rønne, M. E., Tandrup, T., Madsen, M., Hunt, C. J., Myers, P. N., Moll, J. M., Holck, J., Brix, S., Strube, M. L., Aachmann, F. L., Wilkens, C., and Svensson, B. (2023) Three alginate lyases provide a new gut *Bacteroides ovatus* isolate with the ability to grow on alginate. *Appl. Environ. Microbiol.* **89**, e0118523

37. Kim, H. T., Chung, J. H., Wang, D., Lee, J., Woo, H. C., Choi, I. G., and Kim, K. H. (2012) Depolymerization of alginate into a monomeric sugar acid using Alg17C, an exo-oligoalginate lyase cloned from *Saccharophagus degradans* 2-40. *Appl. Microbiol. Biotechnol.* **93**, 2233–2239

38. Jiang, Z., Guo, Y., Wang, X., Li, H., Ni, H., Li, L., Xiao, A., and Zhu, Y. (2019) Molecular cloning and characterization of AlgL17, a new exo-oligoalginate lyase from *Microbulbifer* sp. ALW1. *Protein Expr. Purif.* **161**, 17–27

39. Zeng, L., Li, J., Cheng, Y., Wang, D., Gu, J., Li, F., and Han, W. (2021) Comparison of biochemical characteristics, action models, and enzymatic mechanisms of a novel exolytic and two endolytic lyases with mannuronate preference. *Mar. Drugs* **19,** 76

40. Yang, J., Cui, D., Ma, S., Chen, W., Chen, D., and Shen, H. (2021) Characterization of a novel PL 17 family alginate lyase with exolytic and endolytic cleavage activity from marine bacterium *Microbulbifer* sp. SH-1. *Int. J. Biol. Macromol.* **169**, 551–563

41. Park, H. H., Kam, N., Lee, E. Y., and Kim, H. S. (2012) Cloning and characterization of a novel oligoalginate lyase from a newly isolated bacterium *Sphingomonas* sp. MJ-3. *Mar. Biotechnol. (NY)* **14**, 189–202

43. Yang, X., Li, S., Wu, Y., Yu, W., and Han, F. (2016) Cloning and characterization of two thermo- and salt-tolerant oligoalginate lyases from marine bacterium *Halomonas* sp. *FEMS Microbiol. Lett.* **363**, fnw079

45. Wang, L., Li, S., Yu, W., and Gong, Q. (2015) Cloning, overexpression and characterization of a new oligoalginate lyase from a marine bacterium, *Shewanella* sp. *Biotechnol. Lett.* **37**, 665–671

50. Liu, X. F., Cao, Y., Zhang, H. L., Chen, Y. J., and Hu, C. J. (2015) Complete genome sequence of *Vibrio alginolyticus* ATCC 17749^T^. *Genome Announc.* **3**, e0150014

66. Mathieu, S., Touvrey-Loiodice, M., Poulet, L., Drouillard, S., Vincentelli, R., Henrissat, B., Skjåk-Bræk, G., and Helbert, W. (2018) Ancient acquisition of “alginate utilization loci” by human gut microbiota. *Sci Rep.* **8,** 8075

67. Cheng, W., Yan, X., Xiao, J., Chen, Y., Chen, M., Jin, J., Bai, Y., Wang, Q., Liao, Z., and Chen, Q. (2020) Isolation, identification, and whole genome sequence analysis of the alginate-degrading bacterium *Cobetia* sp. cqz5-12. *Sci Rep.* **10**, 10920

68. Xue, Z., Sun, X. M., Chen, C., Zhang, X. Y., Chen, X. L., Zhang, Y. Z., Fan, S. J., and Xu, F. (2022) A novel alginate lyase: identification, characterization, and potential application in alginate trisaccharide preparation. *Mar. Drugs* **20,** 159

69. Tang, X., Jiao, C., Wei, Y., Zhuang, X. Y., Xiao, Q., Chen, J., Chen, F. Q., Yang, Q. M., Weng, H. F., Fang, B. S., Zhang, Y. H., and Xiao, A. F. (2022) Biochemical characterization and cold-adaption mechanism of a PL-17 family alginate lyase Aly23 from marine bacterium *Pseudoalteromonas* sp. ASY5 and its application for oligosaccharides production. *Mar. Drugs* **20,** 126

70. Kraiwattanapong, J., Ooi, T., and Kinoshita, S. (1997) Cloning and sequence analysis of the gene (alyⅡ) coding for an alginate lyase of *Pseudomonas* sp. OS-ALG-9. *Biosci. Biotechnol. Biochem.* **61**, 1853–1857

71. Dobruchowska, J. M., Bjornsdottir, B., Fridjonsson, O. H., Altenbuchner, J., Watzlawick, H., Gerwig, G. J., Dijkhuizen, L., Kamerling, J. P., and Hreggvidsson, G. O. (2022) Enzymatic depolymerization of alginate by two novel thermostable alginate lyases from *Rhodothermus marinus*. *Front. Plant Sci.* **13**, 981602

72. MacDonald, L. C., Weiler, E. B., and Berger, B. W. (2016) Engineering broad-spectrum digestion of polyuronides from an exolytic polysaccharide lyase. *Biotechnol. Biofuels Bioprod.* **9**, 43

73. Shin, J. W., Lee, O. K., Park, H. H., Kim, H. S., and Lee, E. Y. (2015) Molecular characterization of a novel oligoalginate lyase consisting of AlgL- and heparinase Ⅱ/Ⅲ-like domains from *Stenotrophomonas maltophilia* KJ-2 and its application to alginate saccharification. *Korean J. Chem. Eng.* **32**, 917–924

74. Li, S., Wang, L., Chen, X., Zhao, W., Sun, M., and Han, Y. (2018) Cloning, expression, and biochemical characterization of two new oligoalginate lyases with synergistic degradation capability. *Mar. Biotechnol. (NY)* **20**, 75–86

75. Cote, C. K., Cvitkovitch, D., Bleiweis, A. S., and Honeyman, A. L. (2000) A novel beta-glucoside-specific PTS locus from *Streptococcus* mutans that is not inhibited by glucose. *Microbiology* **146**, 1555–1563

76. Roland, K., Curtiss, R. Ⅲ, and Sizemore, D. (1999) Construction and evaluation of a ∆cya∆crp *Salmonella typhimurium* strain expressing avian pathogenic *Escherichia coli* O78 LPS as a vaccine to prevent airsacculitis in chickens. *Avian Dis.* **43**, 429–441

77. Edwards, R. A., Keller, L. H., and Schifferli, D. M. (1998) Improved allelic exchange vectors and their use to analyze 987P fimbria gene expression. *Gene* **207**, 149–157

78. Kovach, M. E., Elzer, P. H., Steven Hill, D., Robertson, G. T., Farris, M. A., Roop, R. M., and Peterson, K. M. (1995) Four new derivatives of the broad-host-range cloning vector pBBR1MCS, carrying different antibiotic-resistance cassettes. *Gene* **166**, 175–176
